# Investigation on the differentiation of two *Ustilago esculenta* strains - implications of a relationship with the host phenotypes appearing in the fields

**DOI:** 10.1186/s12866-017-1138-8

**Published:** 2017-12-06

**Authors:** Yafen Zhang, Qianchao Cao, Peng Hu, Haifeng Cui, Xiaoping Yu, Zihong Ye

**Affiliations:** Zhejiang Provincial Key Laboratory of Biometrology and Inspection & Quarantine, College of Life Sciences, China Jiliang University, Hangzhou, Zhejiang 310018 China

**Keywords:** *Ustilago esculenta*, *Zizania latifolia*, Differentiation, Pathogenicity, Life cycle, Endophytic

## Abstract

**Background:**

*Ustilago esculenta*, a pathogenic basidiomycete fungus, infects *Zizania latifolia* to form edible galls named Jiaobai in China. The distinct growth conditions of *U. esculenta* induced *Z. latifolia* to form three different phenotypes, named male Jiaobai, grey Jiaobai and white Jiaobai. The aim of this study is to characterize the genetic and morphological differences that distinguish the two *U. esculenta* strains.

**Results:**

In this study, sexually compatible haploid sporidia UeT14/UeT55 from grey Jiaobai (T strains) and UeMT10/UeMT46 from white Jiaobai (MT strains) were isolated. Meanwhile, we successfully established mating and inoculation assays. Great differences were observed between the T and MT strains. First, the MT strains had a defect in development, including lower teliospore formation frequency and germination rate, a slower growth rate and a lower growth mass. Second, they differed in the assimilation of nitrogen sources in that the T strains preferred urea and the MT strains preferred arginine. In addition, the MT strains were more sensitive to external signals, including pH and oxidative stress. Third, the MT strains showed an infection defect, resulting in an endophytic life in the host. This was in accordance with multiple mutated pathogenic genes discovered in the MT strains by the non-synonymous mutation analysis of the genome re-sequencing data between the MT and T strains (GenBank accession numbers of the genome re-sequencing data: JTLW00000000 for MT strains and SRR5889164 for T strains).

**Conclusion:**

The MT strains appeared to have defects in growth and infection and were more sensitive to external signals compared to the T strains. They displayed an absolutely stable endophytic life in the host without an infection cycle. Accordingly, they had multiple gene mutations occurring, especially in pathogenicity. In contrast, the T strains, as phytopathogens, had a complete survival life cycle, in which the formation of teliospores is important for adaption and infection, leading to the appearance of the grey phenotype. Further studies elucidating the molecular differences between the *U. esculenta* strains causing differential host phenotypes will help to improve the production and formation of edible white galls.

**Electronic supplementary material:**

The online version of this article (10.1186/s12866-017-1138-8) contains supplementary material, which is available to authorized users.

## Background

*Zizania latifolia*, also named wild rice, belonging to the Oryzeae of the Gramineae, was one of the six important grain crops in ancient China [1]. Now, it has been documented to be an asexual aquatic vegetable with unique flavour and texture that has been cultivated in East and Southeast Asia for more than 1500 years due to the colonization of *Ustilago esculenta* [2, 3]. *U. esculenta*, which is found in the Ustilaginaceae of the basidiomycetes, is a typical smut fungus related to *Ustilago maydis* [4]. It is widely believed that *U. esculenta* only triggers swollen host stems while completely suppressing host inflorescence development and seed production [5, 6]. Studies demonstrated that *U. esculenta* only grows within the stem of the aquatic grass *Z. latifolia* during plant development and overwinters in the rhizomes that are left in the field after harvest by the farmers to allow for reproduction [7, 8]. The swollen stems (Additional file 1a), which are the real edible part called edible galls, were named “Jiaobai” in China [6], “kambong” in India [8] and “makomotake” in Japan [9]. It was reported that the galls were always observed to carry mature fungal spores in India, and the galls full of spores caused hypersensitivity pneumonitis in Japan [8, 10]. In China, some plants appeared in the fields with galls full of dark-coloured teliospores (Additional file 1b), named “grey Jiaobai”. Those grey Jiaobai should be discarded immediately in fields to ensure yields because only “white Jiaobai” have economic value in China and are counted in the yield. White Jiaobai was named for its white appearance (Additional file 1c) and is a traditional Chinese medicine, though the inner tissues of the edible galls are full of fungal hyphae [11]. Through artificial selection, the plant rhizomes with the desired edible galls have been maintained for rounds of farming. However, grey Jiaobai regularly appears in the fields at rates of nearly 10%. Grey Jiaobai could not be reversibly developed to white Jiaobai. Rather, it eventually becomes “male Jiaobai”, which does not have galls, shows normal flowering, and does not contain *U. esculenta* [12]. It is hypothesized that through continuous artificial selection, white Jiaobai may have developed a special plant-fungus interaction, resulting in edible galls with better nutritional and medicinal value. In contrast, grey Jiaobai, which is induced by the interaction between the plant and its parasitic fungus, seems to be a harmful plant disease. In the fields, if farmers do not remove grey Jiaobai immediately, the incidence rate of grey Jiaobai and male Jiaobai increases in the next rounds of harvest.

It is speculated that the appearance of different phenotypes of Jiaobai in the fields (grey Jiaobai and white Jiaobai) was due to the existence of differentiated strains of *U. esculenta*, which caused diverse host defence responses [5, 7, 13–16]. Yang and Leu [2] were first aware of the strain differentiation and therefore coined the names T (sporidial) type (in the grey galls full of grey teliospores) and M-T (mycelia-sporidial) type (in the white galls with abundant mycelium and little sori) to specify the strains. This nomenclature was used by other researchers [13]. To confirm the differentiation of the strains and their relationship to the formation of white and grey Jiaobai, pairs of haploid heterogametic strains from white Jiaobai (UeMT10 and UeMT46) and grey Jiaobai (UeT14 and UeT55) of the cultivar Longjiao 2^#^ were isolated. Mating assays in vitro and inoculation assays were carried out successfully, along with microscopic tracing of fungal growth *in planta* using modified isolated strains that over-express the nuclear localized *EGFP* gene. Using these methods, the morphology, physiological-biochemical characteristics and pathogenicity of the MT and T strains were observed and compared. Non-synonymous mutations between the MT and T strains were analysed to support the differentiation, which may lead to the two phenotypes of *Z. latifolia* that formed in fields.

## Methods

### Plants, strains isolation and culture

The Jiaobai of the cultivar Longjiao 2^#^ (Variety number: 2,008,024 in vegetable of Zhejiang Province) used in this study was cultivated at the base of Jiaobai in Tongxiang (30°68′87.82N, 120°54′05.49E). Haploid heterogametic strains UeMT10 (CCTCC AF 2015020)/UeMT46 (CCTCC AF 2015021) isolated from white Jiaobai and UeT14 (CCTCC AF 2015016)/UeT55 (CCTCC AF 2015015) isolated from grey Jiaobai of the cultivar Longjiao 2^#^ were used in this study. For selecting these haploid strains, teliospores from swollen stems of grey and white Jiaobai were picked (the tissues of white Jiaobai were kept at 4 °C for more than two weeks to allow the scattered formation of teliospores) and cultured dispersedly on PDA medium (which was bad for mating of *U. esculenta*) to germinate the teliospores. After 24 h, the formed basidiospores were re-suspended in ddH_2_O by washing, and diluted to a concentration of ~10^3^/mL, then picked out by means of single spore culture [17, 18]. 60 single colonies were selected and cultured in pairs of mating-compatible sporidial strains (Additional file 2) on YEPS medium (2% sucrose, 2% tryptone and 1% yeast extract, pH 7.3) with 1% activated charcoal. For detection of mating type genes, primers were used as listed in Additional file 3. Strains were cultured on YEPS medium at 28 °C, 180 rpm.

### Mating experiment

UeMT10, UeMT46, UeT14 and UeT55 were cultured on YEPS solid medium for 2 days separately. Then colonies were picked and re-suspended in YEPS broth, with an OD_600_ of around 1.5. After mixing the strains of UeMT10 and UeMT46 (or UeT14 and UeT55) with equal volumes, 1–2 μL mixture was spotted to YEPS solid medium and inoculated at 28 °C for observations with 12 h intervals.

### Inoculation assay

UeMT10, UeMT46, UeT14 and UeT55 were grown in YEPS medium for 24 h with continuous shaking at 28 °C. Cells were centrifuged and resuspended in infiltration buffer (1% sucrose, 1% tryptone and 0.5% yeast extract, pH 7.3) to an OD_600_ of 2.0. Each combination of two heterogametic strains was done in a ratio of 1:1 for two strains. The mixed sporidia suspension was infiltrated into the internode of the rhizomes that were water cultured for 2 weeks using a 1 mL needleless syringe. Then rhizomes were soaked in the mixed sporidia suspension overnight. Then the rhizomes were transferred to a small container full of soil mixed with the inoculated suspension for 7 days to keep the efficient infection. At last, the inoculated rhizomes were cultivated in a bigger planting box in the glasshouse (Additional file 4).

### Life cycle tracing observation

The pUe-OE plasmid encoding the enhanced green fluorescent protein (EGFP) reporter with a nuclear localization signal was linearized and used to transform the haploid strains as described previously [19]. Four recombinant strains (UeMT10::EGFP-NLS, UeMT46::EGFP-NLS, UeT14::EGFP-NLS, and UeT55::EGFP-NLS) were constructed. Mating experiments and inoculation assays were done with strains capable of transgenic mating in vitro. Samples were collected at 12-h intervals and observed under a fluorescence microscope (Nikon SMZ1500). Following the inoculation assays, teliospores were collected from the plants after tumour formation.

### Teliospore germination assays

Teliospores were collected from grey Jiaobai and advanced age white Jiaobai (kept at 4 °C) of the cultivar Longjiao 2^#^, washed in sterile water, and diluted in YEPS liquid medium. Each of them was coated onto YEPS solid medium and cultured at 28 °C. After 12 h, 24 h and 36 h, they were observed under a light microscope. All samples were analysed in triplicate.

### Growth rate study

Haploid strains were used. The yeast-like fungus was obtained from YEPS solid medium, and a single colony was picked for culture in YEPS liquid medium for 48 h. Cells were centrifuged and re-suspended in YEPS to an OD_600_ of 1.0. A cell suspension of 1 mL in 100 mL of prepared medium with the indicated compounds (different treatments are described below) was incubated and placed on a shaker at 180 r/min at 28 °C. After 12 h, 24 h, 36 h, 48 h, 60 h and 72 h, the absorbance of the cultured solution was read at 600 nm and recorded. In addition, plate colony-counting method was used to measure the cells population. Cultures in each condition collected for A_600_ measurement was diluted to OD_600_ of ~0.1 (the dilution times was recorded D), and 100 uL of the dilutions were introduced to three separate YEPS plates. Colonies were counted 3 days after culture. The number of colonies was recorded as N. Cells population (cfu/mL) was gotten by the formula: 10 × D × N.

### Treatments

Growth rate and mating experiments were carried out on the prepared medium with different treatments. At 12-h intervals, mating colonies were mounted for observation on a microscope.

Media containing different carbon and nitrogen sources were prepared as described as follows: Basic medium (BM) including K_2_HPO_4_ 1 g, MgSO_4_·7H_2_O 0.5 g, FeSO_4_·7H_2_O 0.01 g and KCl 0.5 g was dissolved in 1000 mL of distilled water and autoclaved for sterilization. The nitrogen sources in the medium also changed. Different nitrogen sources (peptone, urea, NH_4_NO_3_, (NH_4_)_2_SO_4_, arginine, methionine or sodium glutamate) were added to BM to make the concentration of nitrogen 20 mmol/L. The medium was then filtered by Millipore filters (0.22 μm), with sucrose as a carbon source (50 mmol/L). Carbon sources were changed in the medium, and the different carbon sources (maltose, lactose, fructose, glucose, galactose, sorbitol or mannitol) were added to BM to reach a concentration of carbon of 50/3 mmol/L. The medium was then filtered by Millipore filters (0.22 μm), with KNO_3_ as the nitrogen source (20 mmol/L). BM with 20 mmol/L KNO_3_and 50 mmol/L sucrose was prepared as the blank control (CK).

All the media had different potential of hydrogen (pH) were prepared as follows: HCl or NaOH was added to the YEPS broth to reach different final pH values (5.3, 6.3, 7.3, 8.3, and 9.3), measured by a pH meter (Starter2100, Ohaus), and then, 1.5% agar was added to obtain solid media, which were autoclaved for sterilization.

Different concentration of H_2_O_2_ were added (the original concentration was 30%) to autoclaved YEPS solid media to obtain different final concentrations of H_2_O_2_ (0.5%, 1.0%, 1.5%, 2.0%, 2.5%, and 3.0%).

### Tissue section staining and microscope observation

Plant samples were collected during the growth periods, cut to pieces, and fixed in 70% (*w*/*v*) ethanol. The morphology of the fungus after inoculation was observed by a fluorescence microscope (Nikon). The tissues of the field samples were stained in a 0.025% (w/v) solution of Aniline blue in lactophenol for 3–5 h, as described in Jose and Louis [20], and then examined by a light microscope (Carl Zeiss Vision Imaging Systems).

### Analysis of non-synonymous mutations between MT and T type strains

Strains were isolated from the Jiaobai cultivar called Longjiao 2^#^. A grinding method was used to isolate the strains. Nine slices from the different samples of Jiaobai (Longjiao 2^#^) were mixed for grinding and then cultured on YEPS media. All the colonies formed after 4 days culture were mixed to isolate the DNA. DNA sequencing and re-sequencing data were both generated using the Illumina HiSeq2000 platform (BGI, Shenzhen, China). The whole genome sequencing results of the MT types isolated from white Jiaobai of the Longjiao 2^#^ variety were deposited on-line (JTLW00000000), and the whole genome re-sequencing of the T types isolated from grey Jiaobai of the Longjiao 2^#^ variety were deposited in GenBank under accession number SRR5889164. Genome Analysis TK (Version 1.6) and SAM tools 1.9 were used to identify the SNPs and indels. InGAP-sv was used to identify the structure variations [21].

### DNA extraction, amplification and sequencing

Genomic DNA of the fungi was extracted by the CTAB method [22]. Fragments were amplified by a standard PCR procedure with primers in Additional file 3. Amplified products were purified by PCR Clean-Up Kit (MOBIO Laboratories) and identified by sequencing.

### Statistical analysis

All experiments were performed in triplicates and data were shown as mean ± SD from three independent experiments. All data were subjected to statistical ANOVA analysis according to the Duncan method, and the probability values of *P* < 0.05 were considered as significant.

## Results

### MT strains showed a growth defect compared to T strains in vitro

**Fig. 1:**
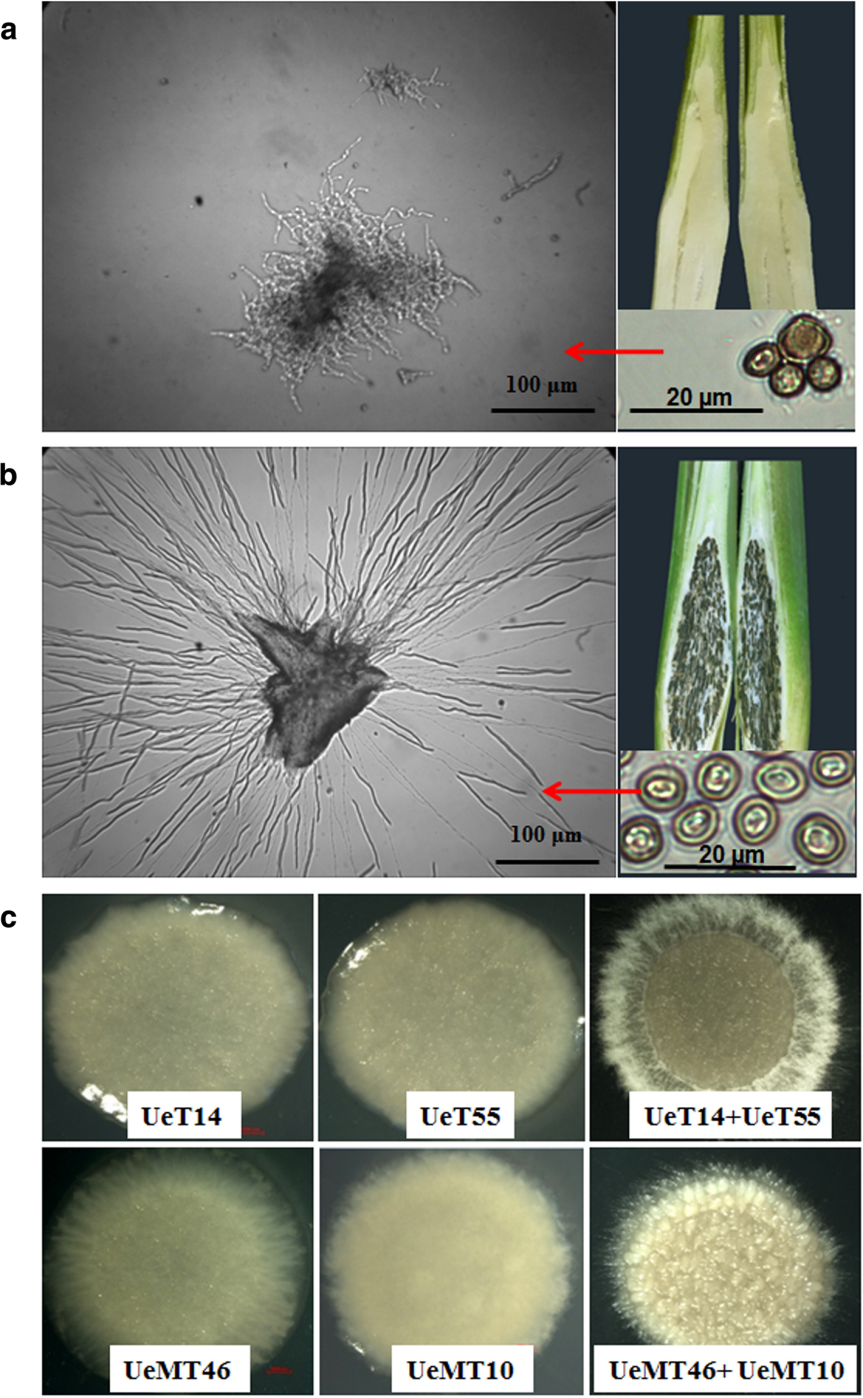
The strains isolated from grey Jiaobai and white Jiaobai. **a** and **b** The teliospores and colonial morphology of MT strain isolated from white Jiaobai (**a**) and T strain isolated from grey Jiaobai (**b**) observed under light microscope. Bars indicated 100 μm (left) and 20 μm (right) respectively. **c** The colonial morphology of UeT14/UeT55, UeMT10/UeMT46 and their mating phenotypes after 3 days’ culture

Sexual reproduction is important for fungal inheritance and variation to enable long-term survival. The haploid sporidium, the meiotic product of basidiomycetes, is ultimately used in sexual reproduction of the fungus [23]. In this study, the haploid sporidia of *U. esculenta* were isolated from the teliospores of the swollen tissues of white Jiaobai (called MT types, Fig. 1a) and grey Jiaobai (called T types, Fig. 1b) of the Longjiao 2^#^ cultivar, and sexually compatible sporidia were isolated. Mating types were identified using in vitro mating experiments (Additional file 2) and by gene sequencing (Additional file 2). Interestingly, none of the isolated T type sporidia tested positive for *a3* or *b3* genes, whereas none of the MT type sporidia carried *a1* or *b1* genes. Two-paired heterogametic haploid strains, UeT14 and UeT55 from grey Jiaobai (called T strains) and UeMT10 and UeMT46 isolated from white Jiaobai (called MT strains), were selected. The four strains were deposited in the China Centre for Type Culture Collection (UeT14: CCTCC AF 2015016, UeT55: CCTCC AF 2015015, UeMT10: CCTCC AF 2015020, UeMT46: CCTCC AF 2015021). Their single strain colonial morphologies or their morphologies after mating are shown in Fig. 1c. Meanwhile, their mating types were identified by PCR, showing that UeT14 was *a1b1*, UeT55 and UeMT46 were *a2b2*, and UeMT10 was *a3b3* (Additional file 2).

**Fig. 2:**
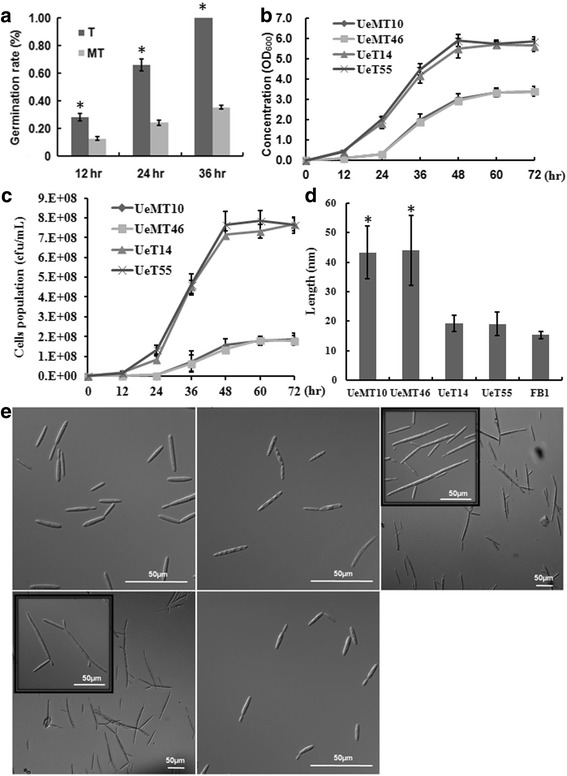
The characteristics of MT and T strains on growth and morphology. **a** The teliospore germination rate of MT and T strains on YEPS solid medium. **b** The growth rate of MT and T haploid strains. **c** Cell population growth curve of MT and T haploid strains. Cells were counted via colony counts. **d** and **e** The micro-morphology and length of haploid strains (UeT14, UeT55, UeMT10, UeMT46 and UmFB1). Microscopic images were taken on a light microscope. Bars indicated 50 μm. The insets images in **e** represent the typical phenotypes of muti-budding

During the sporidia isolation process, we found that many MT type teliospores had not germinated and that the colonial morphology of the MT type was different from that of the T type (Fig. 1a, b) at 3 days after plating on PDA medium, indicating a basic growth difference between them. The growth difference was first shown by a germination rate experiment, which showed that the MT type teliospores had a significantly reduced germination rate that was less than half that of the T type (Fig. 2a; Additional file 5a). Additionally, almost half of the MT type teliospores did not germinate after prolonged cultivation, while the T type teliospores nearly had 100% germination after 36 h (Fig. 2a; Additional file 5a), indicating a defect in the germination ability of the MT type teliospores. We also observed that the T type teliospores did not germinate earlier (Additional file 5a). The growth rates of the sporidia were also compared. The results showed that the logarithmic phase of the MT strains was from 24 h to 48 h, almost 12 h later and shorter than the T strains. Finally, the MT strains reached a lower final optical density and a lower cell population (Fig. 2b, c). However, UeMT10 and UeMT46 showed similar growth rate and phenotype, as did UeT14 and UeT55 (Fig. 2). Using microscopic visualization, we found that both the MT and T strains reproduced by budding; however, the MT strains showed a multi-budding phenotype, and the cell length was longer than those of the T and FB1 strains of *U. maydis* (Fig. 2d, e). The mating capacity was also tested. After mating, hyphae were observed in T strains at 24 h, but the MT type strain hyphae appeared at 48 h (Additional file 6). The T types form normal hyphae with a dense and white appearance in YEPS solid medium, while the MT types showed fewer and shorter hyphae, without obvious white hyphae formation (Fig. 1c, Additional file 6).

### MT and T type strains used different nitrogen and carbon sources

**Fig. 3:**
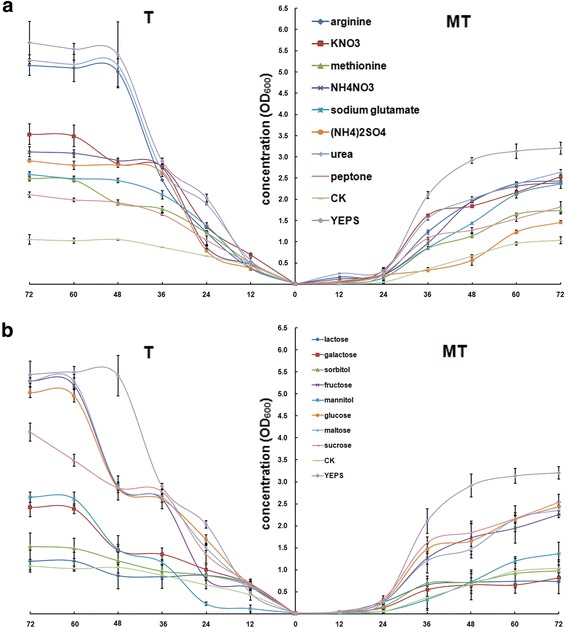
The growth rate of haploid strains under different nitrogen (**a**) and carbon (**b**) sources treatments. Growth rates of the T and MT strains were measured by OD_600_ at an interval of 12 h culture in prepared medium. The BM medium with 20 mm/L KNO_3_ and 50 mm/L as nitrogen and carbon sucroses was prepared as control (CK). The YEPS medium was chosen as nutritious substrate. **a** Different nitrogen source (peptone, urea, NH_4_NO_3_, (NH_4_)_2_SO_4_, arginine, methionine or sodium glutamate) was added to BM medium, with the c (N) = 20 mmol/L. Growth rate of the UeT55 (left) and UeMT46 (right) were measured by OD_600_ at an interval of 12 h culture in the prepared media. **b** Different carbon source (maltose, lactose, fructose, glucose, galatose, sorbitol or mannitol) was added to BM medium, with the c (C_6_) = 100 mmol/L. Growth rate of the UeT55 (left) and UeMT46 (right) were measured by OD_600_ at an interval of 12 h culture in the prepared media

Nitrogen and carbon are the basic growth sources for all living cells and have been reported to be important in a co-existing system [24–26]. We first detected the nitrogen and carbon utilized by the MT and T strains for their growth. Although the cell length and branching were different between the T and MT strains, optical density was chosen as an adequate measurement of their growth rates, based on the fine linear consistency of the optical density value with the sporidia cell number (Additional file 5b). However, the cell number of the T strains was nearly twice that of the MT strains at the same OD_600_ value (Additional file 5b). In the basic medium without nitrogen or carbon sources, the growth status was deficient, but there was a similar final concentration between the MT and T strains with different starting logarithmic growth time points. Additionally, the MT strains obviously grew after 24 h of culture, at least 12 h slower than the T strains (Fig. 3). After adding the individual nitrogen or carbon source, the starting logarithmic growth time point was approximately 12 h for the T strains but was 24 h for the MT strains. In assessing the ability to utilize the exogenous nutrient sources, the results showed a better utilization of the exogenous nutrient sources by the T strains, except with sodium glutamate, lactose and sorbitol in general (Fig. 3). Looking at the nitrogen sources, urea and arginine promoted the growth of the T strains and reached a higher concentration similar to the nutritious YEPS medium. We also noted that, except for (NH_4_)_2_SO_4_, the nitrogen sources could also somewhat accelerate the growth of the MT strains, showing a similar final cell density to the T strains when sodium glutamate or peptone were chosen (Fig. 3a). Looking into the carbon sources, we found that glucose, fructose and maltose were better utilized, while lactose and sorbitol had no effect on the sporidia growth both in the MT and T strains. Sucrose played a better role in the growth of the MT strains, while mannitol and galactose could be utilized by the T strains but not the MT strains (Fig. 3b).

The influence of the distinct nitrogen and carbon sources in stimulating mating response and hyphal growth was also verified. Using stereo microscope observation, the morphogenesis time was calculated, and the hyphal length was measured. Statistical analysis of the results showed that carbon sources had little effect on the promotion or inhibition of mating or hyphal growth, while the nitrogen sources were important (Table 1, Additional file 7). First, inorganic nitrogen sources were bad for *U. esculenta* mating. In particular, NH_4_NO_3_ and (NH_4_)_2_SO_4_ inhibited the mating progress in the MT strains, slowed down the mating speed and decreased the hyphal growth of the T strains. Second, *U. esculenta* preferred organic nitrogen sources for mating. The T strains preferred peptone and urea, while the MT strains preferred arginine, methionine and sodium glutamate. Interestingly, arginine and sodium glutamate showed an obviously inhibitory influence on T strain mating, including a delayed mating time along with fewer hyphae and a shorter hyphal length (Table 1). Through the above research, YEPS medium showed a better effect on the growth rate and mating and, therefore, was chosen for all the experiments in this study (Fig. 3, Table 1).

**Table 1:** Comparision of the mating response and the length of filaments in presence of distinct nitrogen or carbon sources in vitro

	MT			T		
	Mating happened time (h)	Hyphal length (μm)		Mating happened time (h)	Hyphal length (μm)	
BM	–			–		
peptone	60	0	+	48	1890.4 ± 224.4^cd^	+++
urea	60	577.5 ± 61.1^g^	+	48	2123.0 ± 188.9^c^	+++
NH_4_NO_3_	–	0	–	84	348.0 ± 42.6^h^	+
(NH4)_2_SO_4_	–	0	–	72	579.0 ± 23.4^g^	+
arginine	60	2066 .8 ± 147.3^c^	+++	84	377.4 ± 24.8^h^	+
methionine	60	2034.0 ± 188.6^c^	++	48	1574.1 ± 262.2^de^	+++
sodium glutamate	60	2691.0 ± 196.6^b^	+++	84	963.0 ± 114.5^f^	++
CK	60	1164.4 ± 234.0^ef^	+	60	1501.9 ± 207.0^de^	+++
maltose	–	0	–	84	1717.4 ± 125.8^d^	+++
lactose	–	0	–	72	1330.8 ± 125.8^e^	+++
fructose	–	0	–	48	1839.7 ± 144.5^cd^	+++
glucose	84	0	–	72	1596.7 ± 200.0^d^	+++
galactose	84	0	–	72	1104.9 ± 92.4^ef^	+++
sorbitol	84	0	–	72	1090.1 ± 135.6^f^	+++
mannitol	60	1026.7 ± 120.1^f^	+	48	1934.1 ± 173.2^cd^	+++
YEPS	60	1281.6 ± 362.2^ef^	++	36	5727.0 ± 233.8^a^	+++

All the hyphal length data were detected three days after mating assays on the basic solid medium with different kind of nitrogen or carbon sources. The basic medium with 20 mmol/L KNO3 and 50 mmol/L sucrose was prepared as the blank control (CK). The superscript letters showed significant differences of the hyphal length at P < 0.05 level, according to One-Way ANOVA analysis with the Duncan method. Integrated mating ability was analyzed by the hyphal length and amount (see Additional file 7), comparing with the mating status of MT strains on CK medium, e.g., a significant decrease in hyphal length from the contrast (1164.4 ± 234.0) means shorter and a similar or longer hyphal length from the contrast means normal. Therein, “-” means no hyphae formation; “+” means less hyphae or shorter hyphal length, without white aerial hyphae; “++” means normal hyphal amount and length, without white aerial hyphae; “+++” means normal hyphal amount and length, with white aerial hyphae observed

### H_2_O_2_ showed different effects on mating in the MT and T strains

**Fig. 4:**
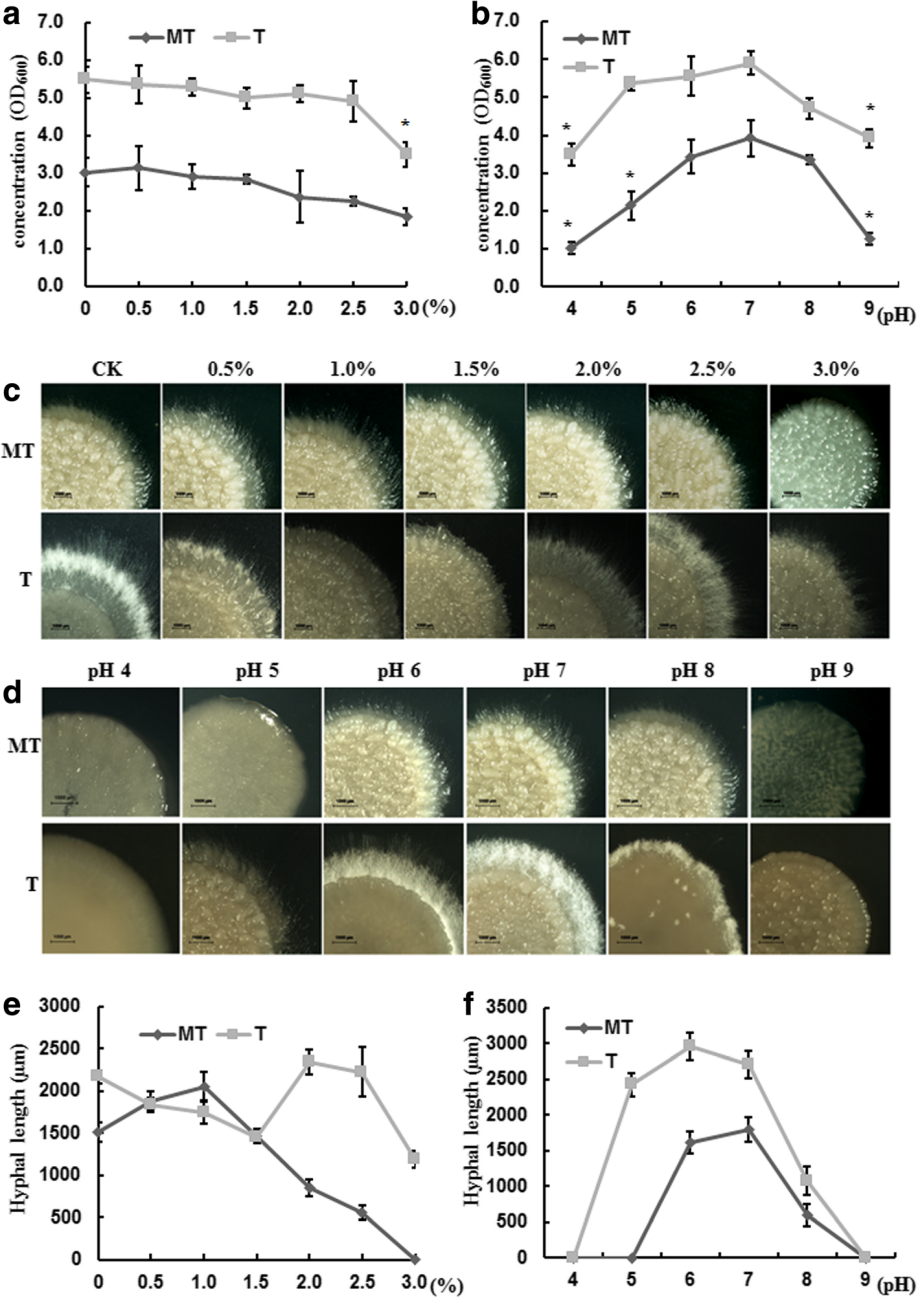
The influence of H_2_O_2_ and pH on the growth of MT and T strains. **a** and **b** The final growth optical density of MT and T strains treated by different concentration of H_2_O_2_ (**a**) and pH (**b**). Samples were collected after 3 days from liquid culture. **c** and **d** The mating phenotype of MT and T strains treated by different concentrations of H_2_O_2_ (**c**) and pH (**d**). Images were taken after 3 days mating by a stereomicroscope. Bars indicated 1000 μm. **e** and **f** The hyphal length of the mated colonies of MT and T strains after 3 days culture when treated with different concentrations of H_2_O_2_ (**e**) and pH (**f**)

The environmental cues H_2_O_2_ and pH were considered to be important in the interaction between pathogens and their host [27, 28]. Therefore, the effects of H_2_O_2_ and pH on the growth and mating of the MT and T strains were studied. There was no obvious difference in the budding growth steps of the MT and T strains in response to these environmental cues, except that the MT type strain had better adaption to a higher concentration of H_2_O_2_ and the T type strain had a wider range adaption to pH (Fig. 4). When the concentration of H_2_O_2_ reached 3.0%, there was a significant reduction in the growth of the T type strain, while the MT type strain did not change compared with the control (Fig. 4a). The growth of the MT type strain was limited when the pH was higher than 8 and lower than 5 (Fig. 4b).

Different concentrations of H_2_O_2_ treatment were carried out in the mating experiments; 3 days later, the mating colonies were observed under the stereo microscope, and the hyphal length was measured. The results are presented in Fig. 4, showing a different induction effect on the MT and T type strains. On the T type strains, hyphal growth was limited from 0 to 2.0%, showing a plummeting hyphal length. However, the hyphal growth was recovered from 2.0% - 2.5% H_2_O_2_, showing a comparable hyphal length to the controls, and then decreased when the concentration was up to 3.0%. Regarding the MT type strains, a low concentration range of 0–1.0% induced hyphal growth, and the hyphal length was longest at 1.0%. Then, the growth was inhibited when the concentration of H_2_O_2_ increased (Fig. 4c, e). Taken together, the effects of different concentrations of H_2_O_2_, which displayed similar reducing tendencies in budding growth of the MT and T strains, could be summarized as being different for the MT and T strains during mating and hyphal growth.

Mixtures of the compatible strains on YEPS solid medium with different pH values were cultured and observed. The results showed that the suitable pH range for both the MT and T strains is pH 6–7. When the pH reached 9, the mating progress stopped both in the MT and T strains. The T strains still can mate and grow at pH 5, indicating a wider range of pH adaption of the T strains during mating progress (Fig. 4d, f).

### The T strains showed a stronger pathogenicity compared to the MT strains in vivo

**Fig. 5:**
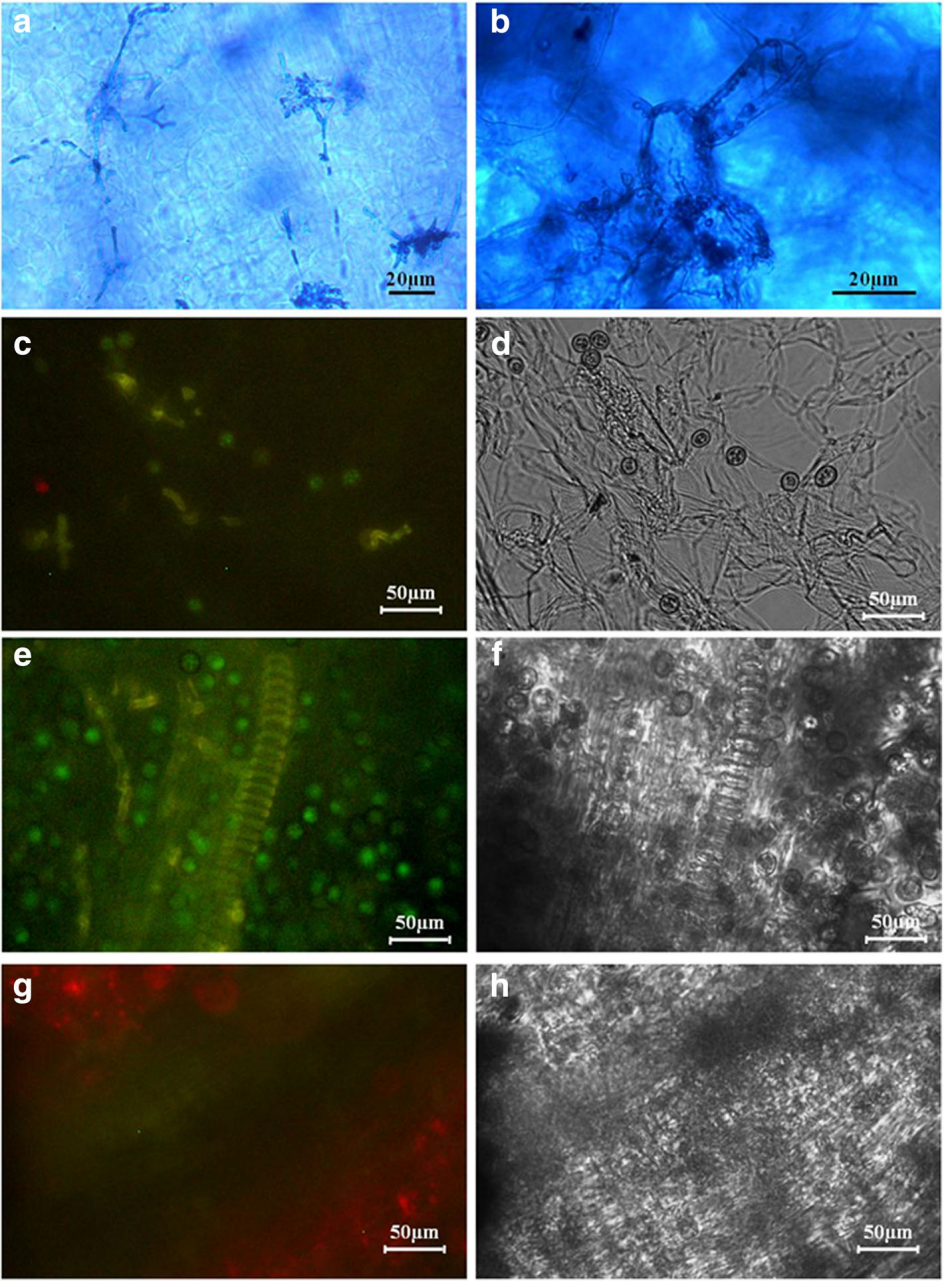
The distribution and morphology of *U. esculenta* in Jiaobai tissues after inoculation. **a** and **b** The fungal morphology in grey Jiaobai (**a**) and white Jiaobai (**b**). The tissues were collected from stem tips of the plants in the fields when they had five leaves, then sliced and stained with aniline blue. Images were taken by light microscope. Bars indicated 20 μm. **c-f** Images were taken by fluorescence microscope. Bars indicated 50 μm. Recombinant strains containing the EGFP reporter gene (UeT14::EGFP-NLS and UeT55::EGFP-NLS) were applied to inoculate. **c** and **d** Samples were collected from the new shoots, 1 month after the inoculation assays. Few spores and hyphae were observed in samples generated by tissue slice. **e** and **f** Samples were collected from the swollen stem of the inoculated plants, nearly 3 month after the inoculation assays. Full of teliospores and hyphae were detected in samples generated by tissue slice. **g** and **h** Samples were collected from wild Jiaobai (the controls) 3 month after the inoculation assays. No fluorescent signal of fungus was detected in samples generated by tissue slice

It was reported that in the natural growth progress, the fungus in grey Jiaobai had a faster infection [7]. In this study, tissue section staining results also indicated the limited infection ability of *U. esculenta* in white Jiaobai. Specifically, at the end of April when the plants grew to five leaves, the fungus could be detected and observed. The grey Jiaobai fungus spread in the parenchymal cells (Fig. 5a), while the white Jiaobai fungus had limited intercellular growth and had a defect in infecting other internal hyphal cells (Fig. 5b).

**Fig. 6:**
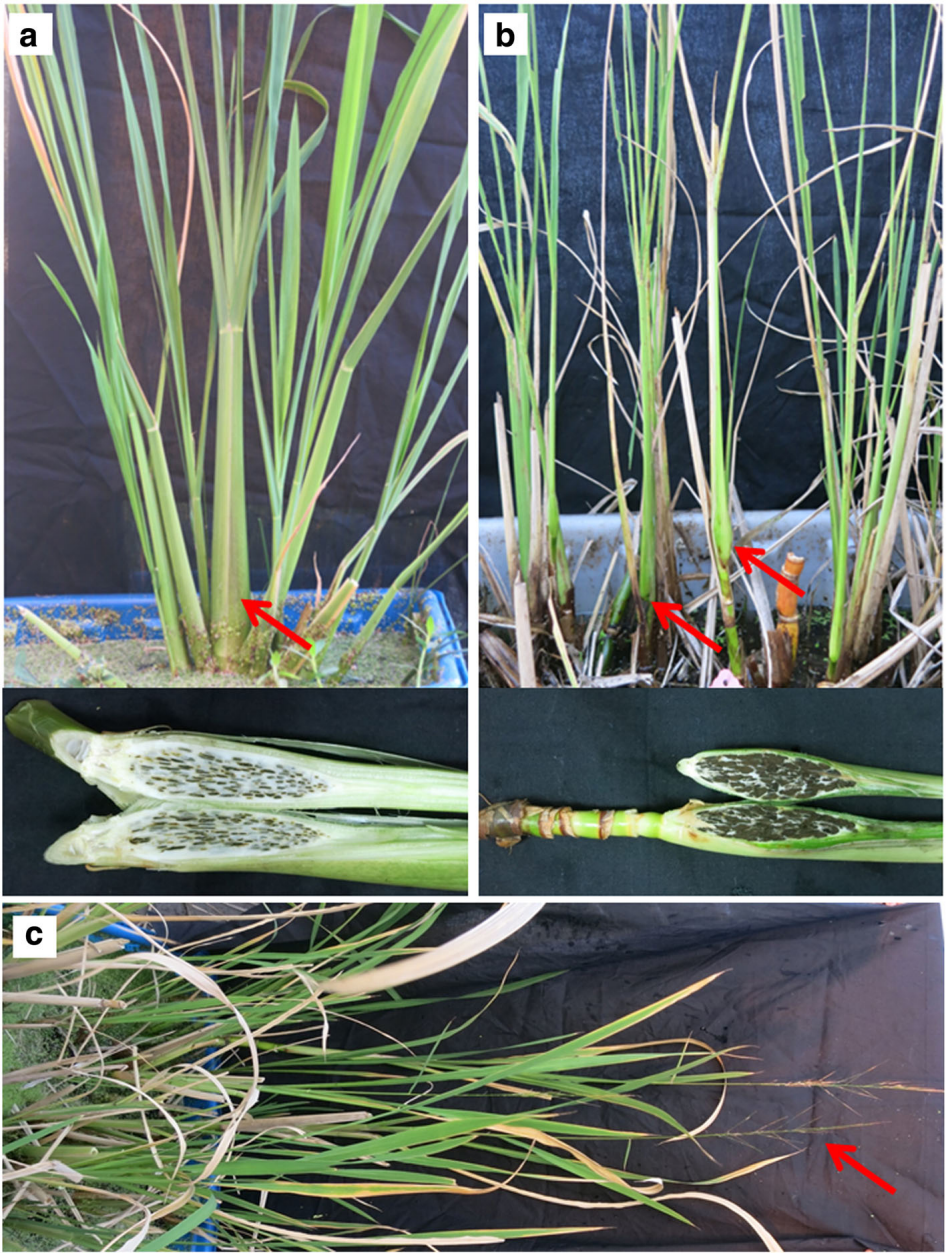
The phenotype of Jiaobao inoculated with different strains and wild Jiaobai. **a** and **b** The swollen stem of Jiaobai after 3 month of the T strains infection (**a**) and the sexual compatible strains respectively from MT and T strains (UeT14 and UeMT46) (**b**), the swollen stem of Jiaobai infected by UeT14 × UeMT10, of UeT55 × UeMT10 was similar to that in b. **c** The wild Jiaobai as control

An inoculated *U. esculenta* system was established, and recombinant strains containing the EGFP reporter gene were applied to distinguish the strains from other fungi inside the plants. As shown in Fig. 5c, fungal structures, including spores and hyphae, were detected in new shoots from the inoculated rhizome and in the topmost internodal region 1 month after T strain infection. Additionally, there were no fluorescent signals detected after inoculation with the MT strains (similar to Fig. 5g). After 3 months, the plants infected with the T strains formed swollen stems full of teliospores (Fig. 5e, f; 6a), while the plants inoculated with the MT strains grew similarly to the control, with normal formation of internodes, nodes and flowering (Fig. 5g, h, 6c). Thus, our results suggested that the MT strains had a defect in infection with the artificial inoculation because the MT strains were absent in the new tissues after inoculation. The defect was not due to mating, as we could see the filaments elongating around the inoculation tissues and mating occurred in the inoculated leaves. Both the T and MT strains were defective in leaf infection but not in mating progress, indicating that only the penetration is related to the defect (Additional file 8).

A cross between the MT and T strains was designed in an additional study. When UeT14 and UeMT10, UeT14 and UeMT46, or UeT55 and UeMT10 were mixed to inoculate the plants, they all showed a delayed infection, and more internodes and nodes formed in the host plants before the stems swelled (Fig. 6b). The galls that formed were all full of teliospores and displayed a grey Jiaobai phenotype (Fig. 6b). Generally, it was considered that the T strains had stronger pathogenicity, which would form grey Jiaobai even when mating with the MT strain during inoculation.

### Comparative analysis of gene mutations in the MT and T strains

To obtain genetic data supporting the conclusion that the differentiation of the MT and T strains is related to the host phenotypes, mixture samples isolated from white Jiaobai (MT types) and grey Jiaobai (T types) of Longjiao 2^#^ were selected, and whole genome re-sequencing was performed. The sequencing results of the MT types and T types were deposited in GenBank under accession number of JTLW00000000 and SRR5889164. SNPs and indels were then identified. Based on this, non-synonymous mutations were analysed between the MT and T types to search for the gene mutations (Additional file 9). Some of the findings were consistent with the physiological diversity that existed between the MT and T strains, especially for the pathogenicity. First, 4438 non-synonymous mutations were detected, 4239 of which were homozygous mutations, indicating a conserved gene structure inside the MT or T type but a large variation between them (Additional file 9). Second, there were 295 mutated genes (with the mutation types of codon deletion or insertion, frame shift, start codon loss, stop codon gain or splice donor or acceptor site change) with coded amino acid changes, representing nearly 6.6% of the total mutations. The remaining 93.4% were base mutations (Additional file 9). Third, 357 of the mutated genes were homologous to the reported functional genes from other fungi, such as *U. maydis*, *Magnaporthe grisea*, and *Candida albicans*. Most of them (310) were pathogenicity-related genes (Additional file 9). Furthermore, 38 of them were not base mutations (confirmed by PCR sequencing of strains isolated from different cultivars), which act on the positive regulation of the fungal virulence reported before in other fungi, and nearly 30% of them resulted in the loss of a start or stop codon or frame shift (see details in Additional file 9). The fact that the genes have amino acid changes does not necessarily mean that their function changed. Actual mutational experimentation needs to be performed to confirm their functions in *U. esculenta* in the future.

## Discussion

*U. esculenta*, an endophytic fungus in *Z. latifolia*, plays important roles in the formation of swollen galls called Jiaobai in China. Two kinds of gall products, called white Jiaobai and grey Jiaobai, occur together during cultivation progress. However, white Jiaobai, which is preferred as a delicacy and in traditional Chinese medicine, is the long-term artificial selection product of constant screening in China. In contrast, grey Jiaobai, which was discarded during every selection round, always appeared in the fields. In this study, the results confirmed that the strains parasitized by white Jiaobai and grey Jiaobai belonged to two distinct types (the MT type and T type) [7, 13, 16], of which the MT type strains showed a growth defect (Fig. 1 and 2), a weaker response to environmental cues (Fig. 3 and 4; Table 1) and a defect in infection (Fig. 5 and 6), along with many functional gene mutations (Additional file 9). For inter-type mating with one strain from the MT type (UeMT10 or UeMT46) and the other from the T type (UeT14 or UeT55), there appeared to be significantly fewer or shorter hyphae than when the T types mated but more and longer hyphae than when the MT types mated (Additional file 6). It appears that the mutations in the MT strains may be recessive, as the T partner appears to compensate for the defect to varying degrees. In addition, when UeT14 is a partner with UeMT10, there appeared to be fewer or shorter hyphae than when it was paired with UeMT46 (Additional file 6). This may be related to the separated *a3* locus that existed in the UeMT10 strain (Additional file 3) or strain variability, which requires further confirmation. The differentiations in the nitrogen or carbon source preference (Table 1) was interesting in that the MT type strains may develop different nutrition requirements and different interactions with the host from T type strains during the long-term endophytic life, similar to some ectomycorrhizal fungi [29]. However, the T type strains, which showed a more comprehensive nutrition preference, seem like a common pathogen that causes the host to form grey Jiaobai, which is common in India [8] and Japan [9]. Interestingly, arginine, a nitrogen source, acts on mating and pathogenicity of dimorphic fungi [30, 31], promoting mating of the MT strains but inhibiting mating of the T strains. In addition, the T strains had a higher hyphal growth at 2–2.5% of H_2_O_2_ than at lower concentrations of 1–1.5%, indicating that H_2_O_2_, as a defence signal in host [32] and also as a signal to be utilized by fungi such as *Botrytis cinerea* for establishment of infection [33], may be exploited by T strains to promote hyphal growth to achieve proliferation. But the real function of H_2_O_2_ in this interaction system needs further studies.

### The defect in the infection of MT strains led to an incomplete life cycle and an absolute endophytic life

In the present study, a similar incomplete life cycle for the T and MT strains in vitro was drawn. Haploid sporidia produced after teliospore germination (Additional file 10a, f) grew exclusively by budding (Additional file 10c, h). When heterogametic sporidia met, mating progress began. Plenty of conjugation tubes formed, and two heterogametic strains mated (Additional file 10d, i). After that, many long hyphae formed, with a vacancy in the middle (Additional file 10e, j), and expanded longer hyphae could be seen around the colonies later (Fig. 1c). So far, they could not form teliospores in vitro even when the culture conditions changed.

In consideration of the fact that *U. maydis*, the closely related species of *U. esculenta*, needs to infect the host to complete its life cycle [34, 35], the inoculated system of *U. esculenta* was established with selected strains. Nuclear green fluorescence T strain teliospores were formed inside the grey galls (Fig. 5c, e).

**Fig. 7:**
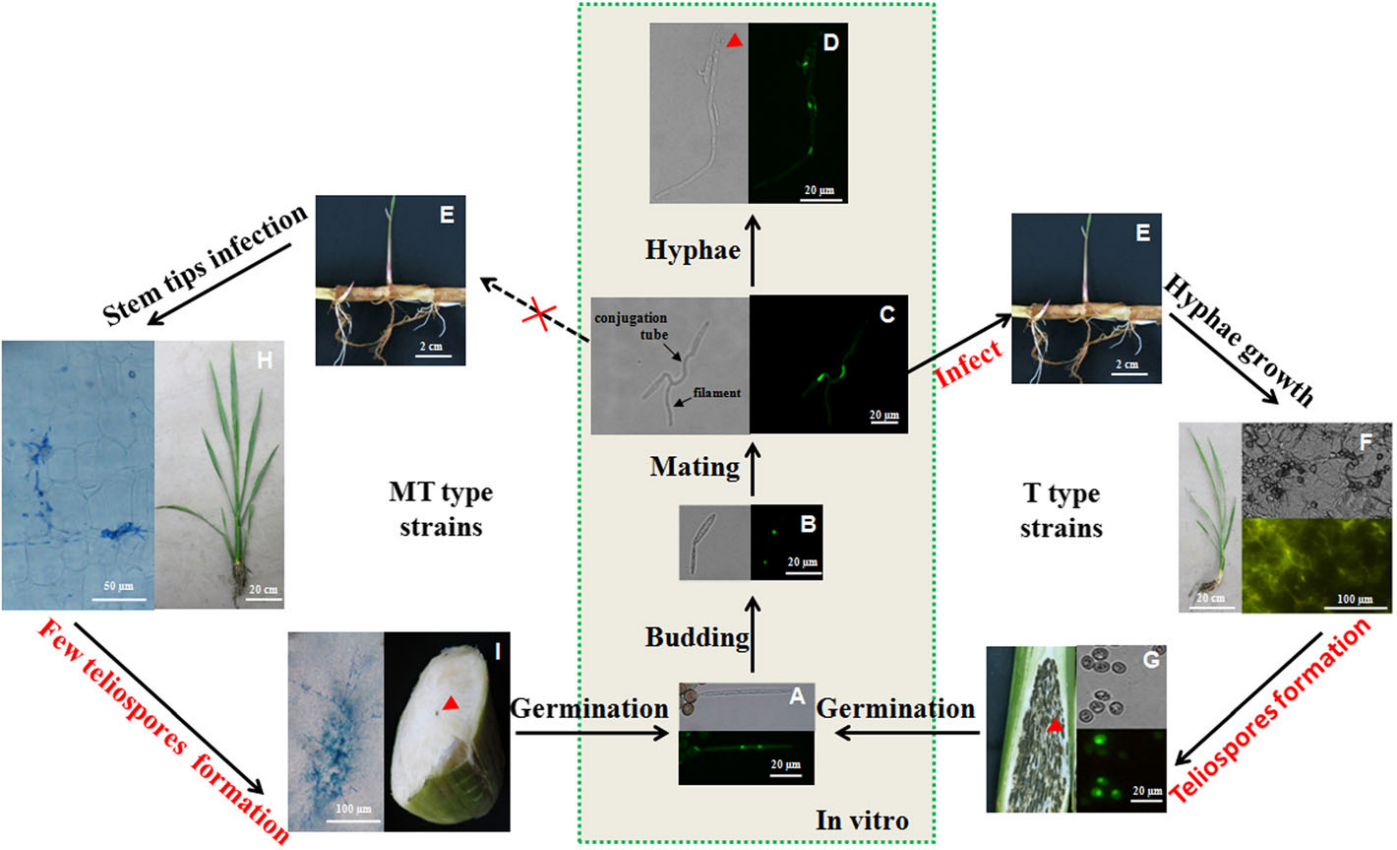
The life cycle of T and MT type strains. **a-d** The progress of teliospore germination (**a**), haploid strain stage (**b**), conjugation tubes formation and mating (**c**), dikaryotic filamentous stage (**d**). Bars indicated 20 μm. The red arrowhead pointed out the vacancy always appeared. **e** The rhizomes of Jiaobai. Bars indicated 2 cm. **f** and **g** The plant inoculated with T type strains. **f** The hyphae and teliospores obviously observed in inoculated plant stem shoots after 1 month. Bars were 20 cm (left) and 100 μm (right). **g** Grey Jiaobai formed (left) and the micromorphology of teliospore inside. Bars were 20 μm. The microscopic images (**a-g**) were taken by a fluorescent microscope. **h** The hyphae growth in white Jiaobai were observed obviously more than 1 month. Bars were 50 μm (left) and 20 cm (right). **i** The swollen stem of white Jiaobai with fewer scattered dark teliospore (right) and the hyphae aggregated inside (left). Bars were 100 μm. **h** and **g** The tissues were stained in aniline blue and microscopic images were taken by light microscope

Therefore, we could draw a complete life cycle of *U. esculenta* (Fig. 7a-g), which began from the germinating stage (Fig. 7a) where the teliospores germinated. After that, sporidia formed from the promycelium and continued budding (Fig. 7b). When two sporidia with different mating types met, they mated. Then, the cytoplast fused through the conjugation tube (Fig. 7c), and a dikaryon formed, which then grew in a certain direction to form the diploid hyphae (Fig. 7d). However, hyphal growth was limited on the medium, and there was no teliospore formation (data not shown). Instead, the mated T strains infected plants (Fig. 7e) with elongated and aggregated hyphae (Fig. 7f). When the plants were 1 month to five leaves, they rapidly produced teliospores, especially in the swollen stem formation (Fig. 7g). Therefore, it was essential for the strains to infect the host, maintain hyphal growth and produce teliospores to live through the adverse situation.

It was confirmed that the MT strains isolated from white Jiaobai lacked the ability to infect the host even when we changed the inoculation conditions (including inoculated tissues\buffer\time, data not shown). Additionally, they formed few teliospores in white Jiaobai with a terrible germination rate (Fig. 2 and 7i), indicating that there was a defect in the infection cycle of the MT strains. Therefore, it was speculated that the MT strains do not share the infection cycle that exists in the T types, which relies on the teliospores to overcome stress and achieve the next infection. The mycelium or fragmented hyphae always existed in the rhizomes, which would be left for the next transplantation after the aboveground culms degenerated and were pruned [8, 36]. It was assumed that the main life cycle of the MT strains was shortened to the asexual reproduction stage, in which only the hyphae were involved. In more detail, the MT strains were colonized first in the rhizomes of the host throughout the year in the forms of mycelium or fragmented hyphae. They propagated fast after colonization to form new shoots, grew through the stem in the right environment and kept growing in the hyphae cluster form (Fig. 7h) in white Jiaobai under cultivation conditions. Sexual reproduction, including teliospore formation (rarely observed in mature late swollen stems, Fig. 7i), teliospore germination, sporidia budding and mating (never found in host tissues and observed in vitro in this study), seemed to be lost in the MT types in the host under long-term acclimation. This was a better explanation for the tillage practice of Jiaobai, in which old rhizomes were left for the next planting round. Otherwise, grey Jiaobai or male Jiaobai (without *U. esculenta* inside) would be widely prevalent.

### Virulence-related genes are mutated in the MT strains to keep a more stable endophytic relationship to the host compared with the T strains

During an infection process, biotrophic fungi must form specific organs, such as appressorium, to access the plant cell wall, where they encounter extracellular surface receptors that recognize pathogen-associated molecular patterns (PAMPs) [37]. PAMPs will initiate PAMP-triggered immunity (PTI) in the host to halt the infection. However, plant pathogens will deliver pathogen protein-like effectors directly into the host plant cells or apoplast to suppress PTI by interfering with recognition or altering resistance response signalling [37]. This would cause effector-triggered immunity (ETI) in a host cultivar that has the capability of recognizing the effector or its action through receptors (resistance gene product). Not surprisingly, pathogens seem to have effectors that are adapted to interfere with PTI, and effectors that trigger ETI are mutated, suppressed, or deleted. In general, successful colonization of the fungus is attributed to the regulation of many pathogenicity-related genes and virulence factors. The comparison of genomic data between the T and MT type strains revealed that many virulence-related genes were mutated in the MT strains. Genes cloned from other cultivated species of Jiaobai in the Zhejiang province also indicated conserved mutations (Additional file 9). Specifically, g3526 (encoding an ATPase), g1901 (encoding a heat shock transcription factor), g3438 and g4493 (belonging to serine/threonine protein kinase genes) possessed codon deletions/insertions. g4697 and g233, encoding gamma-butyrobetaine, 2-oxoglutarate dioxygenase and transposon repressor protein, respectively, had frame shifts. Stop codon mutations occurred in g6458 (encoding transposase) and g3103 (encoding an ATP-binding cassette), which had important roles in the pathogenicity of *M. grisea*. Hence, it was reasonable to speculate that the weaker adaptability to the environment and pathogenicity were related to genetic mutations to give the MT strains a more stable endophytic life in the host. However, actual mutational experimentation needs to be performed in the future.

First, the MT strains with many important mutated virulence factors might lack the related PAMPs or effectors to achieve a fast and successful infection. They cannot spread out in the rhizomes until the plants grow to more than five leaves in white Jiaobai in the fields, while the fungus in grey Jiaobai can grow in the stem and form teliospores when sprouting [3, 36]. Farmers should carefully screen and keep the old rhizomes, where the original MT strains colonized, because white Jiaobai can become grey Jiaobai, but grey Jiaobai can never become white Jiaobai in the fields. All the facts indicated that some mutations in the MT strains interrupted the functional PAMPs or effectors, which might make the host lose the PTI or ETI response, which ensures the opportunistic colonization of the MT strains. In contrast, the T strains caused a plant disease response, which weakens plant germination and growth or induces plant immunity to form male Jiaobai in the fields.

Second, the mutated genes might be related to the growth defect in the MT strains discovered in vitro (Fig. 2), including the defect in teliospore germination and budding cell separation, which might be lost in the MT strains, allowing for a simpler life in the host. Additionally, the defect would prevent the generation of sexual sporidia, which might cause greater genetic variation through sexual reproduction, breaking the stable interaction [38], or mating with other sporidia that exist in the environment, causing disease. Additionally, the sporidia from the T and MT strains could mate with each other using different mating type genes (UeT14 and UeMT10, UeT14 and UeMT46, UeT55 and UeMT10) and achieve a successful infection to generate grey Jiaobai (Fig. 6b). Inversely, the T strains developed a stronger adaptive capacity to the environmental cues, allowing them to seize infection opportunities (Fig. 3 and 4), generate teliospores to resist various kinds of stress in nature, and take advantage of sexual reproduction for adaption to changed circumstances. This could be the main reason for the difficulty in clearing out harmful grey Jiaobai phenotypes in the fields.

In summary, it is reasonable to assume that the MT strain pathogenicity defect allowed it to keep a stable entophytic life in the host, which never impacted host growth, except for flowering. In contrast, the T strains behave as a pathogen and cause grey Jiaobai, resulting in short plants with a low germination rate [15]. Therefore, it was believed that the MT strains may develop a closer relationship to the host and a different mechanism to interact with the host to avoid the plant defence response. Details of the interaction mechanism need to be further studied.

## Conclusion

Sexually compatible haploid sporidia UeT14/UeT55 from grey Jiaobai (T strains) and UeMT10/UeMT46 from white Jiaobai (MT strains) were isolated, and in vitro mating and inoculation assays were successfully established in this study. The study provides a detailed demonstration that the MT type strains isolated from white Jiaobai were different from the T type strains isolated from grey Jiaobai. In general, the MT strains displayed a growth and infection defect and were more sensitive to external signals. They have multiple mutated genes, especially genes homologous to pathogenicity-related genes. Furthermore, the endophytic life of the MT strains was shortened to only an asexual reproduction stage, in which only hyphae were involved. However, the T strains showed a complete survival life cycle, in which teliospore formation was important for adversity adaption and the next infection, leading to a host stem that was full of teliospores. These findings indicate that the differentiation of the *U. esculenta* strains plays an important role in developing their host phenotypes. However, further studies elucidating the molecular differences between the *U. esculenta* strains need to be performed to explain the exact mechanism of the formation of the edible white galls.

## Additional files

Additional file 1: The phenotypes of grey Jiaobai and white Jiaobai in the fields. **a** The swollen stem of Jiaobai pointed by red arrowhead. **b** The grey Jiaobai full of dark-colored teliosorus. **c** The white Jiaobai with white inner tissues. (JPEG 1847 kb)

Additional file 2: Selection and confirmation of the haploid strains. **a** Sexual compatible sporidia were confirmed by mating assays with each two of sporidia isolated mixed and observation of filament formation. **b** The map of *a* loci found in *U. esculenta*. Imaginary line indicated separated area. **c** The map of *b* locus found in *U. esculenta*. **d** The mating type gene of the obtained four haploid strains confirmed by PCR that UeT14 was *a1b1*, UeT55 and UeMT46 were *a2b2*, and UeMT10 was *a3b3*. The accession numbers of the *a* and *b* mating type genes were follows: KT343769 for *mfa3.2*, KT343770 for *pra3*, KT343771 for *mfa3.1*, KT343772 for *mfa1.2*, KT343773 for *mfa1.3*, KT343774 for *pra1*, KT343775 for *mfa2.3*, KT343776 for *mfa2.1*, KT343777 for *pra2*, KU056861 for *bE1*, KU056862 for *bE2*, KU056863 for *bE3*, KU056864 for *bW1*, KU056865 for *bW2*, KU056866 for *bW3*. Marker was DL 2000. (JPEG 1059 kb)

Additional file 3: Primers used in this study. (DOCX 17 kb)

Additional file 4: The inoculation procedure. **a** The Jiaobai seedlings developed from rhizomes of wild Jiaobai to at least 3 leaves. **b** The mixed sporidia suspension was infiltrated into the internode of the rhizomes with a syringe with needle. **c** The whole inoculated rhizomes were soaked in the mixed sporidia suspension overnight (about 12 h). **d** The inoculated rhizomes were transferred to a small container full of soil mixed with the inoculated suspension for 7 days. **e** The inoculated rhizomes were cultivated in a bigger planting box in the glasshouse at 28 °C. (JPEG 1254 kb)

Additional file 5 The germination of teliospores and the relationship with cells population and the absorbance of sporidia suspension. **a** MT and T types of teliospores germinated after 12 h, 24 h, 36 h. Bars indicated 50 μm. **b** sporidia were colony cultured on YEPS solid medium, and picked to culture in YEPS liquid medium for 48 h. 5 mL of Cells were re-suspended with 100 mL YEPS liquid medium to culture for 8 h. All the cells were then centrifuged and re-suspended with YESP liquid medium with gradient concentration measured by OD_600_ (0, 0.5, 1.0, 1.5, 2.0, 2.5, 3.0, 3.5, 4.0, 4.5, 5.0, 5.5 or 6.0). The cells population of each cell resuspension with different OD_600_ were measured by colony counts. The linearity for cells population and the absorbance of sporidia suspension was established. (JPEG 1266 kb)

Additional file 6: The growth status and hyphae formation of mixed strains with different mating type genes observed under stereomicroscope**.** Mating assays with sexual compatible strains including UeT14 x UeT55, UeT14 x UeMT46, UeT14 x UeMT10, UeT55 x UeMT10 and UeMT46 x UeMT10, and sexual imcompatible strains UeT55 x UeMT46 were carried out. The hyphae formation and growth conditions were compared at 24 h, 36 h, 48 h and 60 h during mating progress. Images were taken by a stereomicroscope. (JPEG 1529 kb)

Additional file 7: The influence of distinct nitrogen and carbon sources stimuli on MT and T strains. **a** Mating phenotypes of MT and T strains on YEPS solid culture medium with distinct nitrogen and carbon sources. Images were taken after 3 days by stereomicroscope. Bars indicated 1000 μm. **b** The hyphal length of MT and T strains colonies treated by distinct nitrogen and carbon sources; The basic medium with 20 mm/L KNO_3_ and 50 mm/L sucrose was prepared as control (CK). (JPEG 1314 kb)

Additional file 8: The growth morphology of *U. esculenta* during infection.** a** and **b** The fungal morphology around the inoculated stem tissues. **c**-**f** The fungal hyphae on the surface of the plant leaves were observed after inoculated with the sexual compatible T strains (**c** and **d**) and MT strains (**e** and **f**). Images were taken by a fluorescent microscope. a, c and e were under fluorescence light. b, d and f were under the white light. Bars indicated 15 μm. (JPEG 1369 kb)

Additional file 9: List of the non-synonymous mutations, 38 of the structural changed pathogenicity related genes and the number of statistical mutated genes in mutation types and functional types. Strains were isolated from the cultivar of Jiaobai called Longjiao 2^#^. Grinding method was used to isolate the strains, and all the colonies formed were mixed to do the sequencing. The sequencing results of the MT types isolated from white Jiaobai were deposited at GenBank under accession JTLW00000000 and the GenBank accession of sequencing data of the T types isolated from grey Jiaobai was SRR5889164. All the non-synonymous mutations were compared between T and MT types, the results listed in Sheet 1. Also, 38 of the structural changed pathogenicity related genes were found out and listed in Sheet 2. These 38 genes were PCR confirmed from 6 cultivars of Jiaobai in Zhejiang Province (Longjiao 2^#^, Zhejiao 2^#^, Zhejiao 6^#^, Suozjiao, 911, Yujiao 3^#^). The number of statistical mutated genes in mutation types and functional types in MT types were listed in Sheet 3. (XLSX 1616 kb)

Additional file 10: The morphology of UeT14::EGFP-NLS strain and its mating progress with UeT55::EGFP-NLS strain**. a** and **f** The germination of T strain teliospore containing EGFP-NLS. **b** and **g** The morphology of UeT14::EGFP-NLS strain; **c** and **h** The UeT14::EGFP-NLS strain reproduced by budding. **d** and **i** Plenty of conjugation tubes formed and the two heterogametic strains mated. **e** and **j** Many long hyphae formed, with a vacancy (red arrowhead point) in the middle. Images were taken by a fluorescent microscope. Bars indicated 20 μm. (JPEG 1266 kb)

## Abbreviations

BM: Basic medium
EGFP: Enhanced Green Fluorescent Protein
H_2_O_2_: Hydrogen peroxide
pH: Potential of Hydrogen
SNP: Single Nucleotide Polymorphism
