# Supplementary figures and images for: Investigation on the differentiation of two Ustilago esculenta strains - implications of a relationship with the host phenotypes appearing in the fields
Source: BMC Microbiol. 2017 Dec 6;17:228. doi: 10.1186/s12866-017-1138-8 (PMC5719756; doi:10.1186/s12866-017-1138-8)

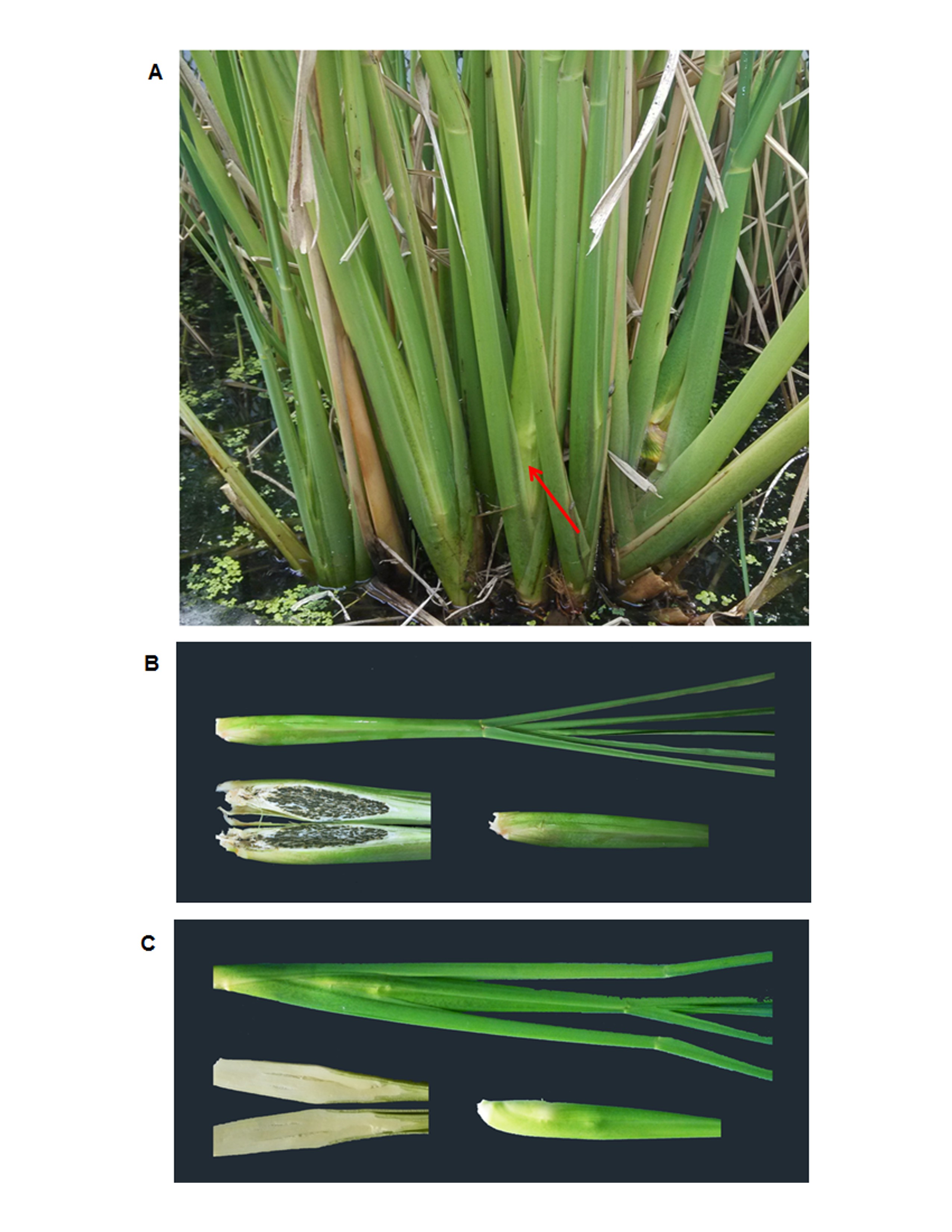

Supplement: Supplementary file 1 — The phenotypes of grey Jiaobai and white Jiaobai in the fields. a The swollen stem of Jiaobai pointed by red arrowhead. b The grey Jiaobai full of dark-colored teliosorus. c The white Jiaobai with white inner tissues. (JPEG 1847 kb) [file 12866_2017_1138_MOESM1_ESM.jpg]

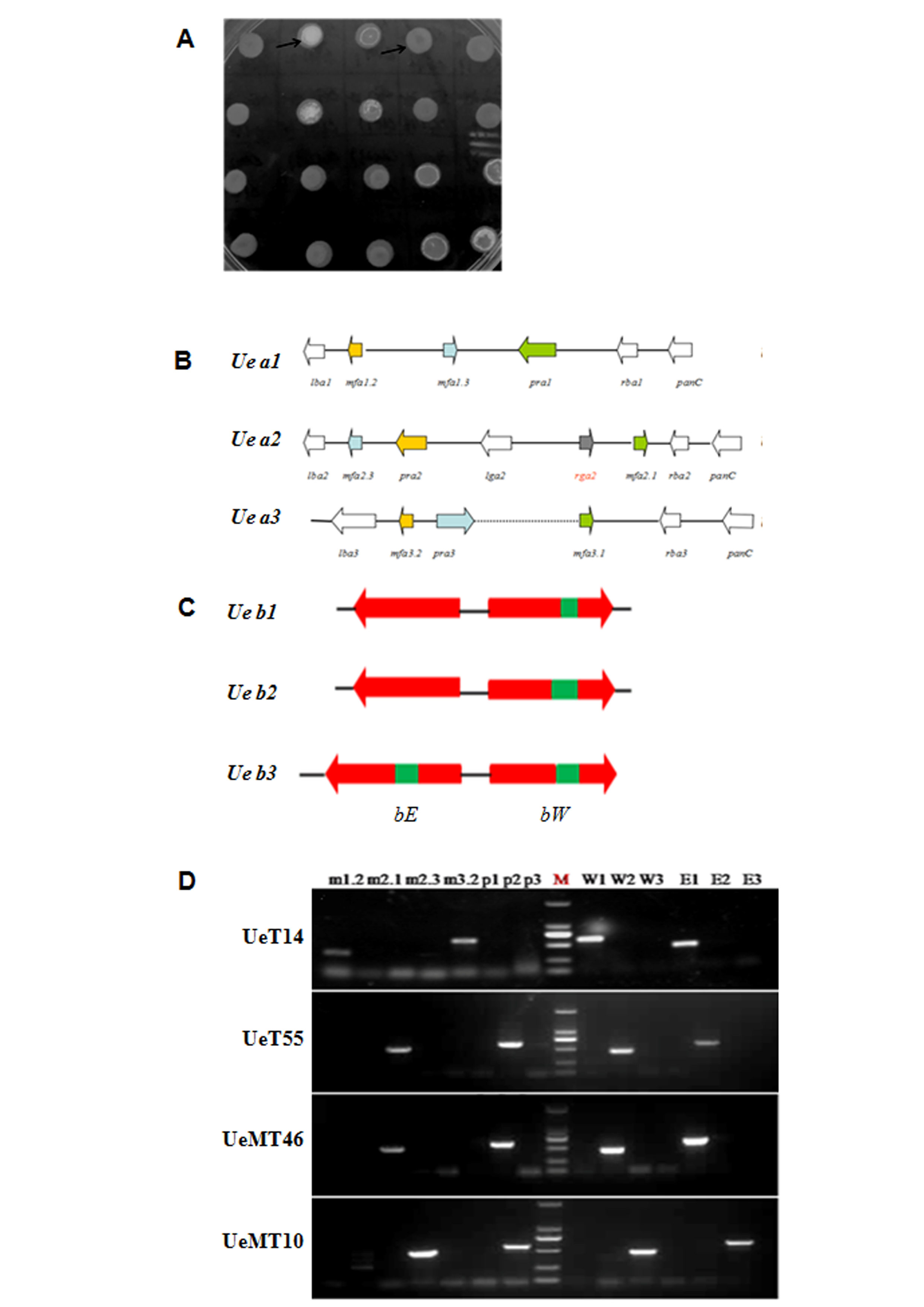

Supplement: Supplementary file 2 — Selection and confirmation of the haploid strains. a Sexual compatible sporidia were confirmed by mating assays with each two of sporidia isolated mixed and observation of filament formation. b The map of a loci found in U. esculenta. Imaginary line indicated separated area. c The map of b locus found in U. esculenta. d The mating type gene of the obtained four haploid strains confirmed by PCR that UeT14 was a1b1, UeT55 and UeMT46 were a2b2, and UeMT10 was a3b3. The accession numbers of the a and b mating type genes were follows: KT343769 for mfa3.2, KT343770 for pra3, KT343771 for mfa3.1, KT343772 for mfa1.2, KT343773 for mfa1.3, KT343774 for pra1, KT343775 for mfa2.3, KT343776 for mfa2.1, KT343777 for pra2, KU056861 for bE1, KU056862 for bE2, KU056863 for bE3, KU056864 for bW1, KU056865 for bW2, KU056866 for bW3. Marker was DL 2000. (JPEG 1059 kb) [file 12866_2017_1138_MOESM2_ESM.jpg]

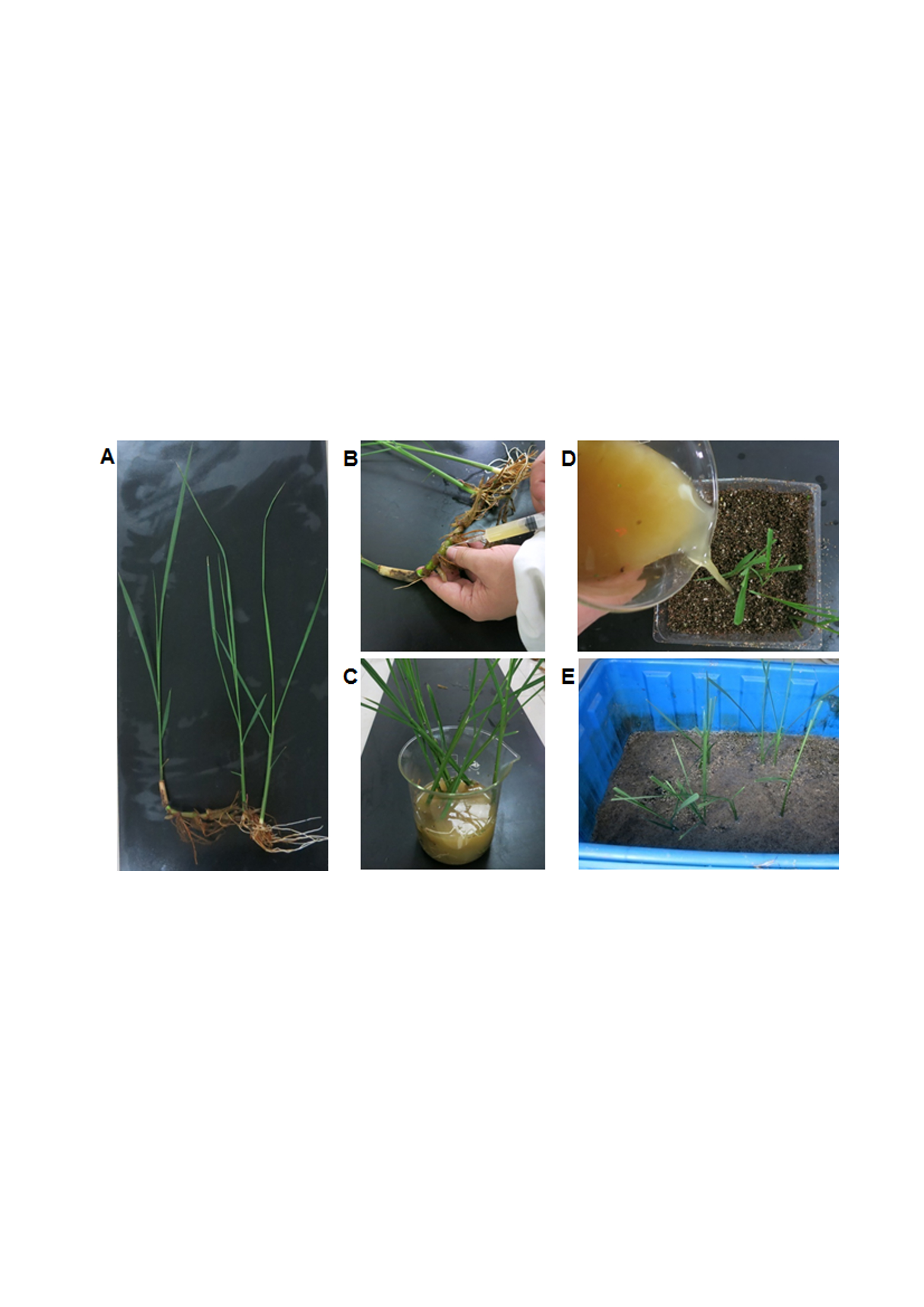

Supplement: Supplementary file 4 — The inoculation procedure. a The Jiaobai seedlings developed from rhizomes of wild Jiaobai to at least 3 leaves. b The mixed sporidia suspension was infiltrated into the internode of the rhizomes with a syringe with needle. c The whole inoculated rhizomes were soaked in the mixed sporidia suspension overnight (about 12 h). d The inoculated rhizomes were transferred to a small container full of soil mixed with the inoculated suspension for 7 days. e The inoculated rhizomes were cultivated in a bigger planting box in the glasshouse at 28 °C. (JPEG 1254 kb) [file 12866_2017_1138_MOESM4_ESM.jpg]

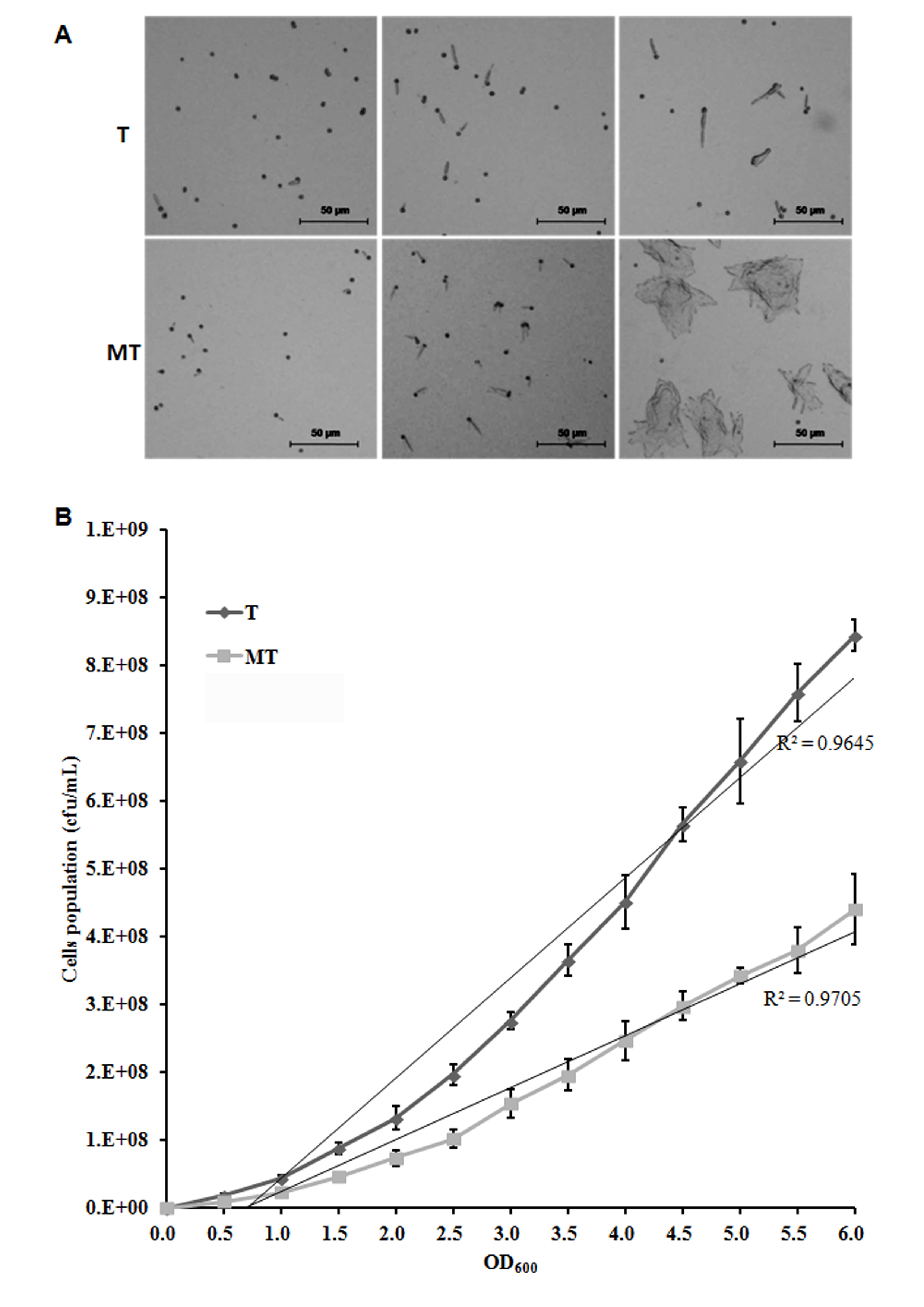

Supplement: Supplementary file 5 — The germination of teliospores and the relationship with cells population and the absorbance of sporidia suspension. a MT and T types of teliospores germinated after 12 h, 24 h, 36 h. Bars indicated 50 μm. b sporidia were colony cultured on YEPS solid medium, and picked to culture in YEPS liquid medium for 48 h. 5 mL of Cells were re-suspended with 100 mL YEPS liquid medium to culture for 8 h. All the cells were then centrifuged and re-suspended with YESP liquid medium with gradient concentration measured by OD600 (0, 0.5, 1.0, 1.5, 2.0, 2.5, 3.0, 3.5, 4.0, 4.5, 5.0, 5.5 or 6.0). The cells population of each cell resuspension with different OD600 were measured by colony counts. The linearity for cells population and the absorbance of sporidia suspension was established. (JPEG 1266 kb) [file 12866_2017_1138_MOESM5_ESM.jpg]

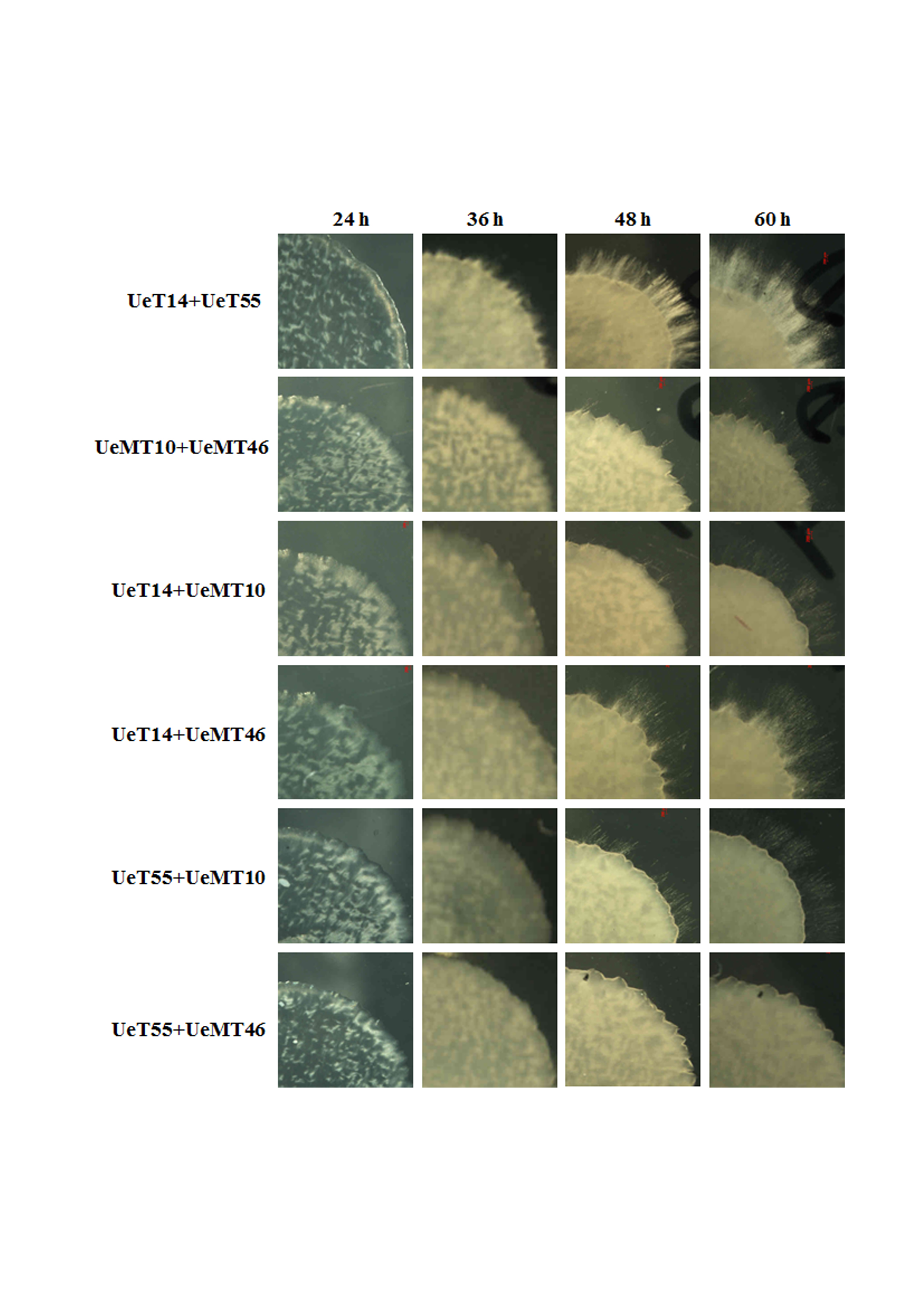

Supplement: Supplementary file 6 — The growth status and hyphae formation of mixed strains with different mating type genes observed under stereomicroscope. Mating assays with sexual compatible strains including UeT14 x UeT55, UeT14 x UeMT46, UeT14 x UeMT10, UeT55 x UeMT10 and UeMT46 x UeMT10, and sexual imcompatible strains UeT55 x UeMT46 were carried out. The hyphae formation and growth conditions were compared at 24 h, 36 h, 48 h and 60 h during mating progress. Images were taken by a stereomicroscope. (JPEG 1529 kb) [file 12866_2017_1138_MOESM6_ESM.jpg]

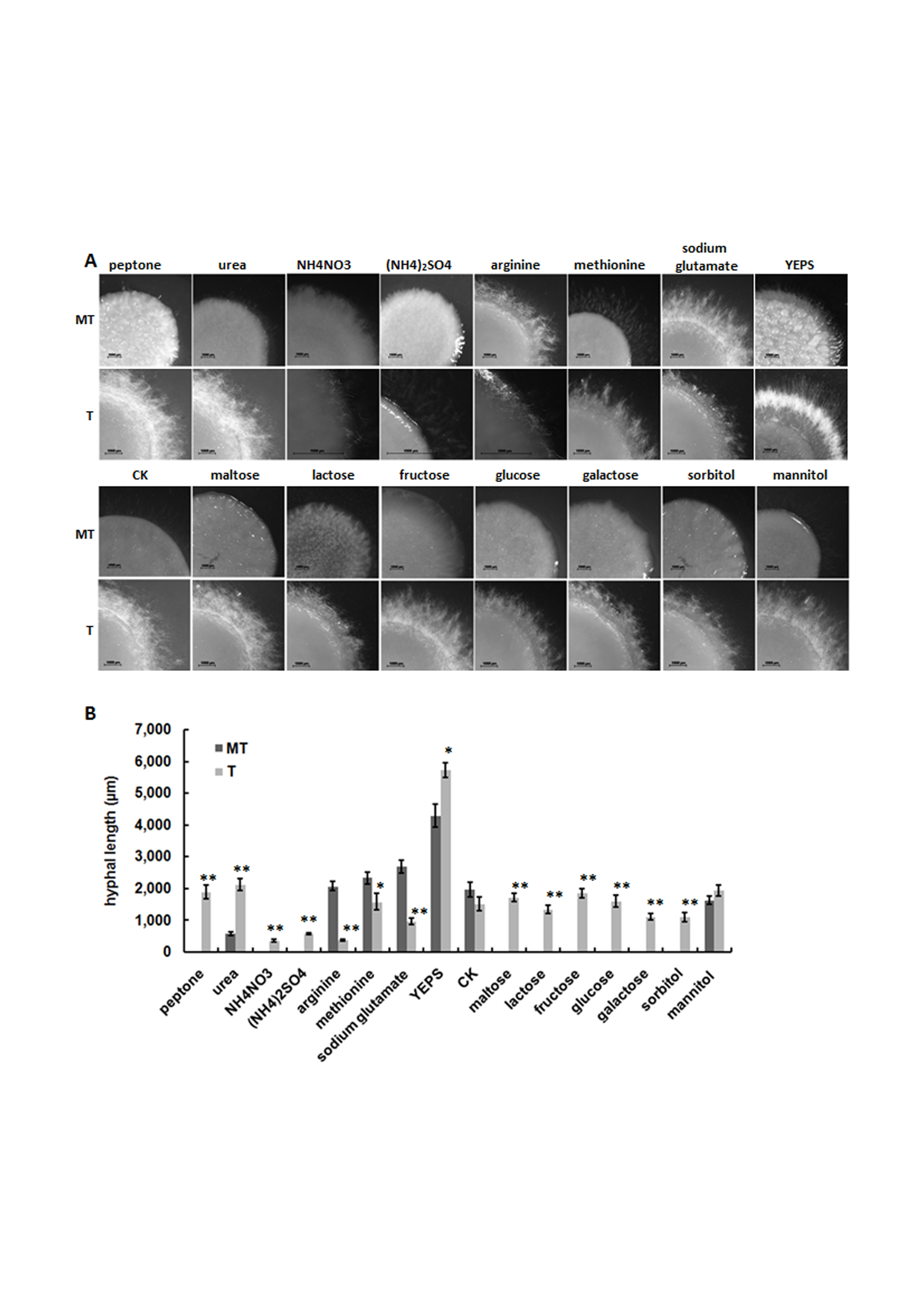

Supplement: Supplementary file 7 — The influence of distinct nitrogen and carbon sources stimuli on MT and T strains. a Mating phenotypes of MT and T strains on YEPS solid culture medium with distinct nitrogen and carbon sources. Images were taken after 3 days by stereomicroscope. Bars indicated 1000 μm. b The hyphal length of MT and T strains colonies treated by distinct nitrogen and carbon sources; The basic medium with 20 mm/L KNO3 and 50 mm/L sucrose was prepared as control (CK). (JPEG 1314 kb) [file 12866_2017_1138_MOESM7_ESM.jpg]

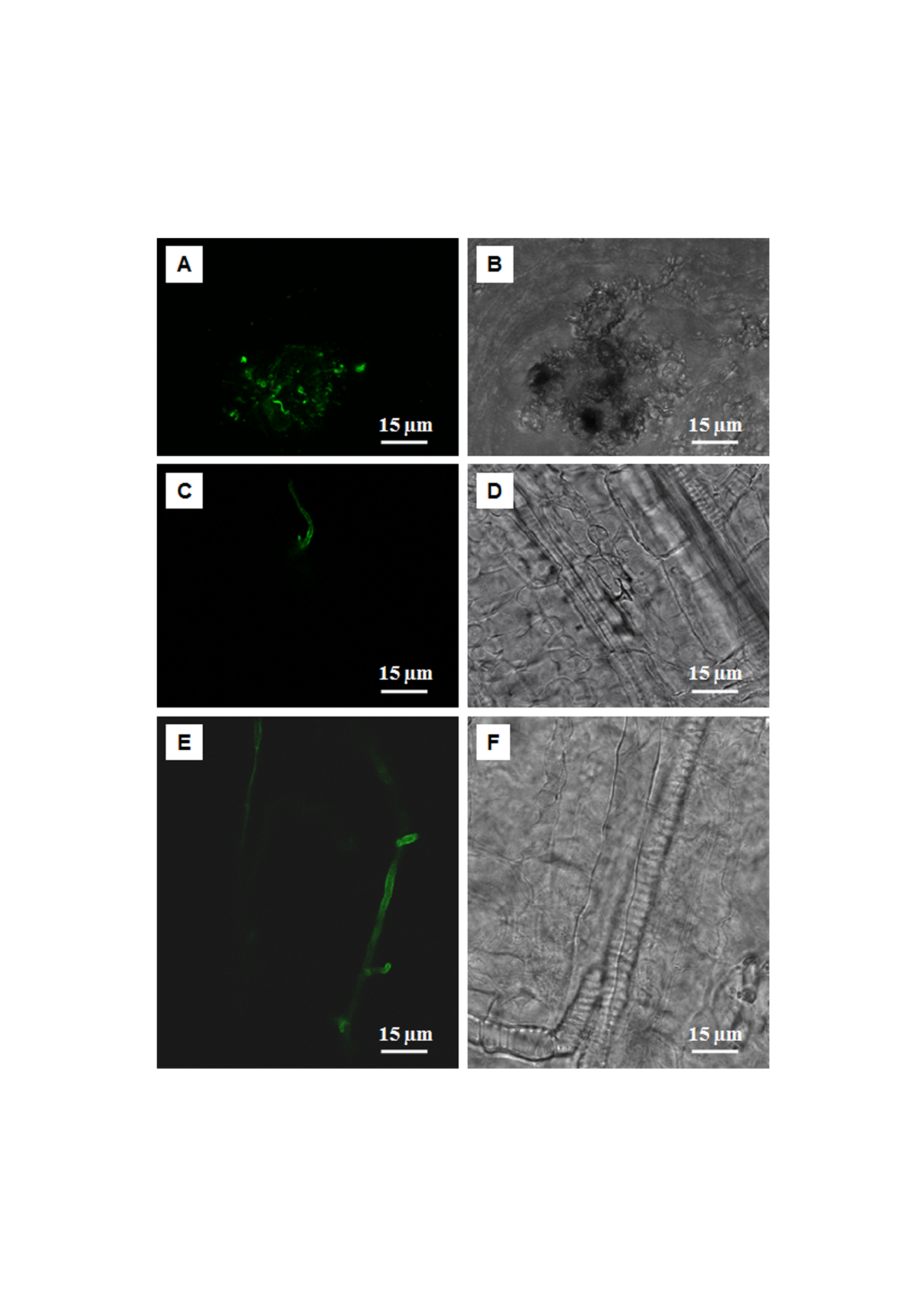

Supplement: Supplementary file 8 — The growth morphology of U. esculenta during infection. a and b The fungal morphology around the inoculated stem tissues. c-f The fungal hyphae on the surface of the plant leaves were observed after inoculated with the sexual compatible T strains (c and d) and MT strains (e and f). Images were taken by a fluorescent microscope. a, c and e were under fluorescence light. b, d and f were under the white light. Bars indicated 15 μm. (JPEG 1369 kb) [file 12866_2017_1138_MOESM8_ESM.jpg]

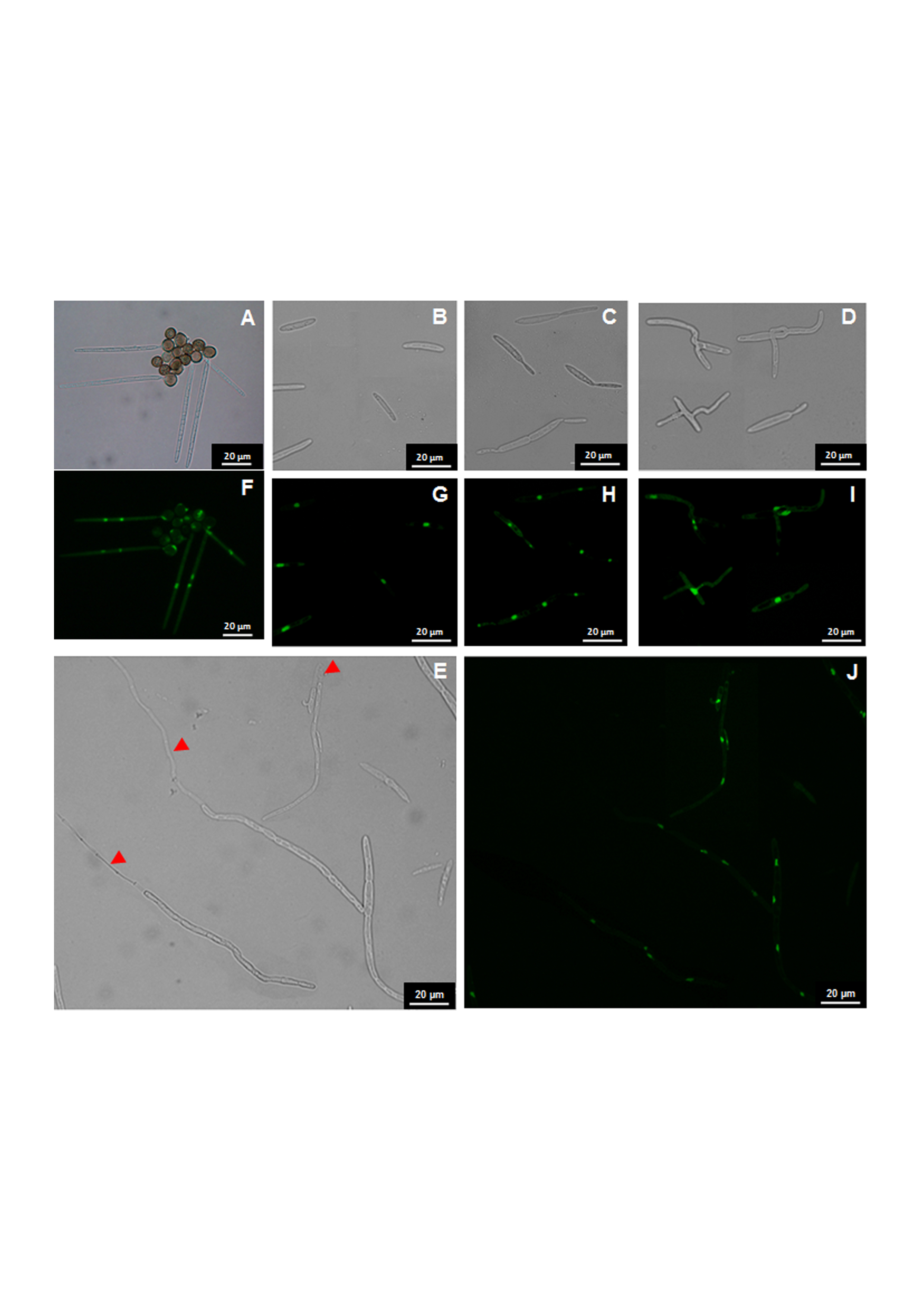

Supplement: Supplementary file 10 — The morphology of UeT14::EGFP-NLS strain and its mating progress with UeT55::EGFP-NLS strain. a and f The germination of T strain teliospore containing EGFP-NLS. b and g The morphology of UeT14::EGFP-NLS strain; c and h The UeT14::EGFP-NLS strain reproduced by budding. d and i Plenty of conjugation tubes formed and the two heterogametic strains mated. e and j Many long hyphae formed, with a vacancy (red arrowhead point) in the middle. Images were taken by a fluorescent microscope. Bars indicated 20 μm. (JPEG 1266 kb) [file 12866_2017_1138_MOESM10_ESM.jpg]
