# Supplementary material for: Investigation on the differentiation of two Ustilago esculenta strains - implications of a relationship with the host phenotypes appearing in the fields
Source: BMC Microbiol. 2017 Dec 6;17:228. doi: 10.1186/s12866-017-1138-8 (PMC5719756; doi:10.1186/s12866-017-1138-8)
Supplement: Supplementary file 3 — Primers used in this study. (DOCX 17 kb) [file 12866_2017_1138_MOESM3_ESM.docx]

**Supplemental Table 1. Primers used in this study.**

| **Gene name** | **Forward primer (5'→3')** | **Reverse primer (5→3')** |
| --- | --- | --- |
| *Pra1* | ATCGGCATCCTCGCTCATTATG | TGCATGCTTGATCTCCGTTGCG |
| *Pra2* | ACAGCACGCTTCCCACCTTTTC | GACAAAGCAGCAGTGAACTGCC |
| *Pra3* | CACAATTCCCATCACGGTGCTC | GAGCGAGAGCACTGATGGAAAG |
| *mfa2.1* | CATCTCGAGTAACCCCTGAACA | GAGATAATCAAAGGTGGGCGGG |
| *mfa2.3* | CCCGGGGATCCTCTAGAGATTA | GAAACGACGAGTTGAGATAATC |
| *mfa1.2* | CACAATGCCCTTTATCTGATGG | GTTGTGGTCAGGCTAACTAGGG |
| *maf3.2* | GAACTAAATCAGAGACGGCTGC | ACGCAAGTCGAGTAGCCGAGAG |
| *bW1* | CAAATCCAATCCATGGTAGCCG | CTGACAGCCGTAATCGAAGTTG |
| *bW2* | CAAGAATGTTCACGCCTTAGCC | CTTGTACCATTCGAGTGGATGC |
| *bW3* | CAGCACTTCTCCTCTTGTCGAG | TGATCAGCAGAGACATCGAGAG |
| *bE1* | CAACTCCTCGAACTACTCACTG | CGTCCAACTTGTGAGTCAGAAC |
| *bE2* | GGAACTAAGCAAGCCTTTTCGG | TCCAGTGCGGATAGGAATGTTG |
| *bE3* | ACCTTCTCCTTCGCCGATTTAC | TTCAGAACTGAGGGAAGAGGTC |
| *g352* | ATGTCCCGCTCCTCCAAGTC | CTACATCAGACTGCTGCGCG |
| *g1901* | AGCATTGGAGGTGCGGATTT | GACGAGTTGGGATGTGAGTG |
| *g4493* | CCTCCATGTACTCCATCACC | ACTGGTCTGCCCAAAGAATG |
| *g3438* | CTACCGACAAGTTCTTCCTC | CCTCGCTATTCTCACTATGC |
| *g4697* | CAGTCTTCTTCCACTACGGC | GGCTTGGTTCTCGACATCACTC |
| *g233* | ATGCGAGCGTGGAAGCGTGG | CTAGTTGGGGATACGGTCGT |
| *g6458* | TTAAGGAGGCCTTAAACTC | TGCCCTTTGGGTTATGC |
| *g3103* | ATGACCGTGACCACGAGCCGC | TTAGCGGTTGCTCTGGTCGT |
